# Directional drift in biologically meaningful vector planes: A proposed geometric framework for early detection of subthreshold disease

**DOI:** 10.1371/journal.pone.0353723

**Published:** 2026-07-30

**Authors:** Gaurav Prakash

**Affiliations:** Department of Ophthalmology, University of Pittsburgh School of Medicine, Pittsburgh, Pennsylvania, United States of America; Commonwealth Scientific and Industrial Research Organisation, AUSTRALIA

## Abstract

**Background:**

Most conventional diagnostic systems rely on fixed thresholds to differentiate disease states from normal. However, early pathological changes may begin before these thresholds are crossed. Therefore, a system that works in this pre-threshold state can potentially lead to earlier diagnosis.

**Objective:**

To propose and evaluate a geometric framework that models early disease as a directional drift from a physiological plane to a pathological plane, allowing for pre-threshold detection using biologically interpretable variables.

**Methods:**

In this modeling study on synthetic data derived from published clinical trends, two clinically meaningful variables were used to define a 2D feature space. The model was applied to a synthetic dataset of 4000 eyes divided into four phenotypes: normal stable (NS), early disease stable (ED_S), early disease progressive (ED_P), and pre-threshold progressive (PT_P). A physiological plane was constructed using range-normalized values from the NS group. A canonical disease vector was derived from the ED_P group. Each subject's follow-up data was transformed into a subject-specific drift vector, and the Composite Drift Score (CDS) was calculated as the product of directional alignment (Directional Emphasis Multiplier, DEM) and a Magnitude-to-Noise Ratio (MNR).

**Results:**

CDS increased significantly over follow-ups in both ED_P and PT_P, distinguishing them from the two stable cohorts (p < 0.001). DEM and MNR components showed consistent trends, with progressive cases exhibiting higher alignment with the disease vector and supra-noise magnitude of change. Visual and statistical analyses confirmed early drift detection even within numerically normal ranges.

**Conclusion:**

In this early modeling study based on simulation data, we could quantify the directional drift with a unitless, interpretable metric (CDS) and its derivatives. It showed similar trends in pre-threshold groups as early disease groups, showing potential for further evaluation.

## Introduction

Over the past few decades, there have been significant improvements in diagnostic technology, particularly in imaging and laboratory methods. This has allowed clinicians to detect increasingly subtle physiological variations, even in individuals without overt disease [1–5]. Despite these capabilities, many diagnostic systems continue to depend on fixed thresholds to define disease. This approach can overlook earlier physiological changes that precede those cutoffs [6–14].

Due to their underlying design, these threshold-based, cut-off style tests tend to perform well in structured datasets where subjects are clearly categorized as either normal or diseased. However, in real-world clinical settings, normal and diseased cases often share overlapping characteristics across multiple parameters. [6,7,13,14].

Futhermore, oversights with multivariable threshold style testing can lead to redundancy and overfitting and reduced diagnostic accuracy [15–23].

**Fig 1:**
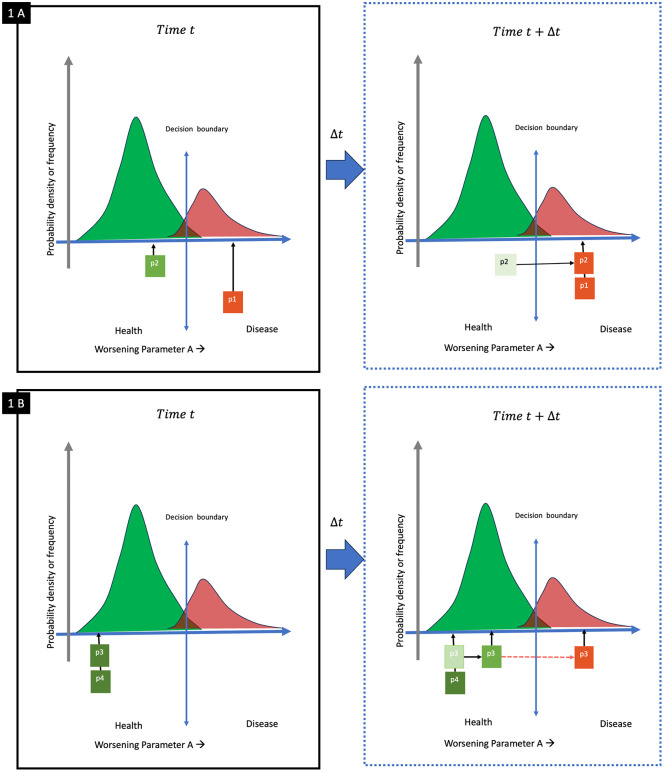
The trend missed in threshold-based tests. **1A.** Two patients in the disease zone may appear clinically similar at a single timepoint despite very different trajectories leading up to disease. P1 is stable and P2 is worsening. **1B.** Two subjects in the normal zone may be on very different paths. P4 is stable but P3 was worsening.

While threshold-based tests are useful for categorizing disease at a single point in time, they cannot account for how a subject reached that point (Fig 1A, 1B). For example, in Fig 1A, patient P1 remains stable and above the threshold (disease zone) at both t and t+Δt, while P2 worsens but only crosses the threshold at the second time point. Despite their very different disease dynamics (P1 has stable disease and P2 has worsening disease), both appear similar if only the current value is considered. Also, deviation away from normal state and progression of worsening often begins well before any statistical cutoffs are breached. For example, Fig 1B shows two more patients, P3 and P4, coming for their first-ever visit at time t. Both are within the so-called normal range currently. However, P3 has been drifting steadily toward the disease state from before, while P4 is stable. A static threshold cannot distinguish between them, failing to detect the early directional trend in P3. Early pathological drift begins when the subject or the organ is still numerically within the accepted values for ‘normal’ population distribution. This phenomenon has been documented in many chronic progressive diseases [24–28].

Clinicians have long recognized that normal range, sub-threshold rising values (more than the reference change value), can be concerning [29–31]. However, it has been challenging to formally characterize or quantify this type of change using traditional tools.

Worsening this issue is the assumption that physiological parameters follow Gaussian distributions, which is often not accurate [7,32–34]. Cross-sectional cutoffs are further complicated by changes in how these scores, or numerical thresholds are defined: as clinical panels revise cutoffs, the decision boundary itself moves one way or the other [30,35].

One way to circumvent these issues is to detect drift, a directional deviation away from the normal state.

To explore the potential of using drift as an alternate vantage point, we should first review natural fluctuations (to due homeostasis) and observation-related noise and formalize our definitions with reference to this study.

Healthy physiological systems maintain a stable state through active regulation resulting in natural fluctuations. Homeostasis is the organism’s tendency to maintain this state. *Physiological variation* can therefore be defined as *homeostatic* self-correction oscillations occur within bounded ranges. [36–38]. *Observation variation,* on the other hand, arises due to multiple reasons within the device-operator-interpreter system. This occurs whenever a measurement is attempted on a biological system or structure [39–46]. *Observation variation*, like *physiological variation,* is typically non-directional and self-correcting. For example, diurnal variations in serum glucose or intraocular pressure (IOP), or postural changes in blood pressure (BP), are expected and healthy [36–38]. Similarly, when measuring IOP, the intra-measurement variation arising from the tonometer, operator experience, and protocol introduces an observation effect that is random and self-correcting, not directional [39–46].

Physiological and observational variation can interact stochastically. There, may not be a clear rule to discriminate them from each other. Therefore, it may be mathematically useful to club them into a single entity, ‘noise’.

This is different from an actual movement aligned with disease -like worsening, which we term *Drift.* Many chronic progressive diseases involve continued cellular deterioration long before diagnostic thresholds are crossed [47–55]. Let us assume that a patient presents to the clinic for annual health checkup and has an HbA1c level of 5.5%, This will be classified as non-diabetic and therefore reassuring. However, if this value rose from 4.5% over a single year, the rate of change may exceed expected biological variability despite remaining below the diagnostic cutoff. Similarly, in keratoconus, an increase in maximum keratometry (Kmax) from 42.0 to 44.5 Diopters may not raise concern because it has not yet crossed the conventional threshold of 47.2D, yet the directional trend is clinically meaningful. This concept extends into multiple clinical domains: in glaucoma, retinal nerve fibre layer thinning may precede visual field loss by years while intraocular pressure remains within the normal range [36]; in chronic kidney disease, directional changes in eGFR and albuminuria signal progressive nephron loss before staging thresholds are crossed [49]; and in Alzheimer's disease, amyloid and tau biomarker trajectories diverge from normal years before cognitive thresholds are breached [24,25]. Across these domains, the common thread is that disease progression is directional and accumulative, and therefore potentially detectable, long before any single numerical cutoff is crossed.

Therefore*, Drift* can be defined as a slow, directional deviation away from this physiological corridor aligned with the expected direction of pathology progression. This occurs before conventional diagnostic thresholds are crossed. It is not defined by a single measurement but by a trajectory over time. This is often still within the so-called “normal” numerical range. [47–55].

It can be argued that medicine, in essence, is systems biology viewed through the lens of pathology. In systems biology, it is well recognized that transitions in system behavior are often preceded by early warning signals. These signs include increased variance, autocorrelation, or critical slowing down, where recovery from oscillations becomes delayed [56–59]. These signals suggest that a system is approaching a tipping point, after which a spontaneous return to the original state becomes unlikely.In our keratoconus example above, the subject trending from 42.0 to 44.5 Diopters over follow-up was demonstrating this Drift, numerically normal, yet directionally meaningful. This concept of drift from a new post-tipping baseline extends beyond keratoconus; in post-transplant settings, directional changes in endothelial cell density and corneal thickness have been shown to predict outcomes such as graft survival [54,55].

**Fig 2:**
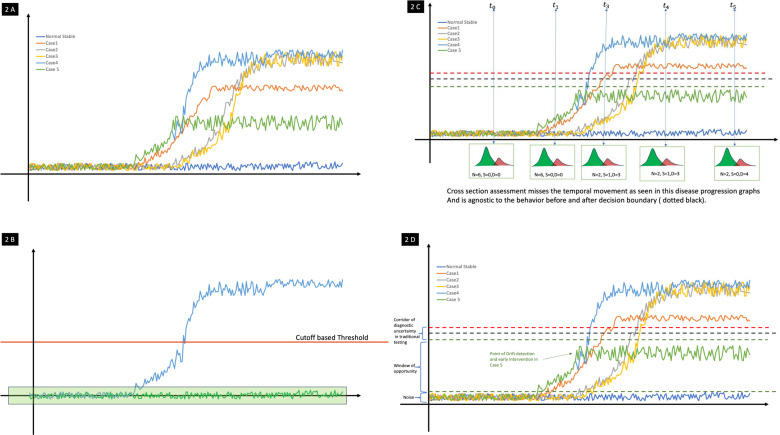
A–D. Temporal patterns of directional drift in simulated subjects. **2A.** Six representative cases showing variable progression and stability. **2B**. Comparison of noise corridor vs disease trajectory. **2C**. Timepoint-overlaid cross-sectional views demonstrating missed progression (in the distribution plots, N = normal, S = suspect, D = Disease) **2D**. Conceptual “window of opportunity” for early detection and intervention.

Therefore, evidence, as outlined above, suggests homeostasis and drift state are not similar, following different trajectories and presentations. These two states therefore appear to be governed by different sets of rules which can be potentially utilized to identify them. The simulated temporal and trajectory-based behavior of 6 representative cases (including one ‘stable normal’) illustrates this further in Fig 2A-2D.

In this visualization, we show six representative simulated cases: one stable normal and five with varying disease trajectories (Fig 2A). These illustrate the diverse ways patients can drift from physiological equilibrium. Some progress gradually, some abruptly, and some not at all. Highlighting the contrast between normal noise (green zone) and true disease drift (blue curve) can suggest how directional movement outside the noise corridor marks meaningful change even before the threshold breach (Fig 2B). Cross-sectional views can obscure these temporal patterns. Fig 2C demonstrates this by comparing overlaid distribution plots at t₀ to t₅. Despite clear progression in several cases, static thresholds at single time points miss the bigger picture. This creates a zone of clinical opportunity: a region of directional drift where early intervention could alter trajectory (Fig 2D). Case 5 in particular shows how an early flag could precede and prevent threshold-defined diagnosis. This case becomes stable after early pre-conventional threshold diagnosis and treatment.

As we have discussed earlier, traditional diagnostic frameworks assume that normal and disease lie along a shared linear axis. This implies that progression is merely a matter of movement along a single continuum [6,7,60,61]. It is a mathematical requirement for comparison and is therefore convenient [60–62]. Threshold-based models assume that a variable’s behavior is interpretable within a known distribution. However, when variables come from fundamentally different biological states, such as a regulated homeostatic system compared to a decompensating one such as the drift, this assumption becomes sub-optimal.

**Fig 3:**
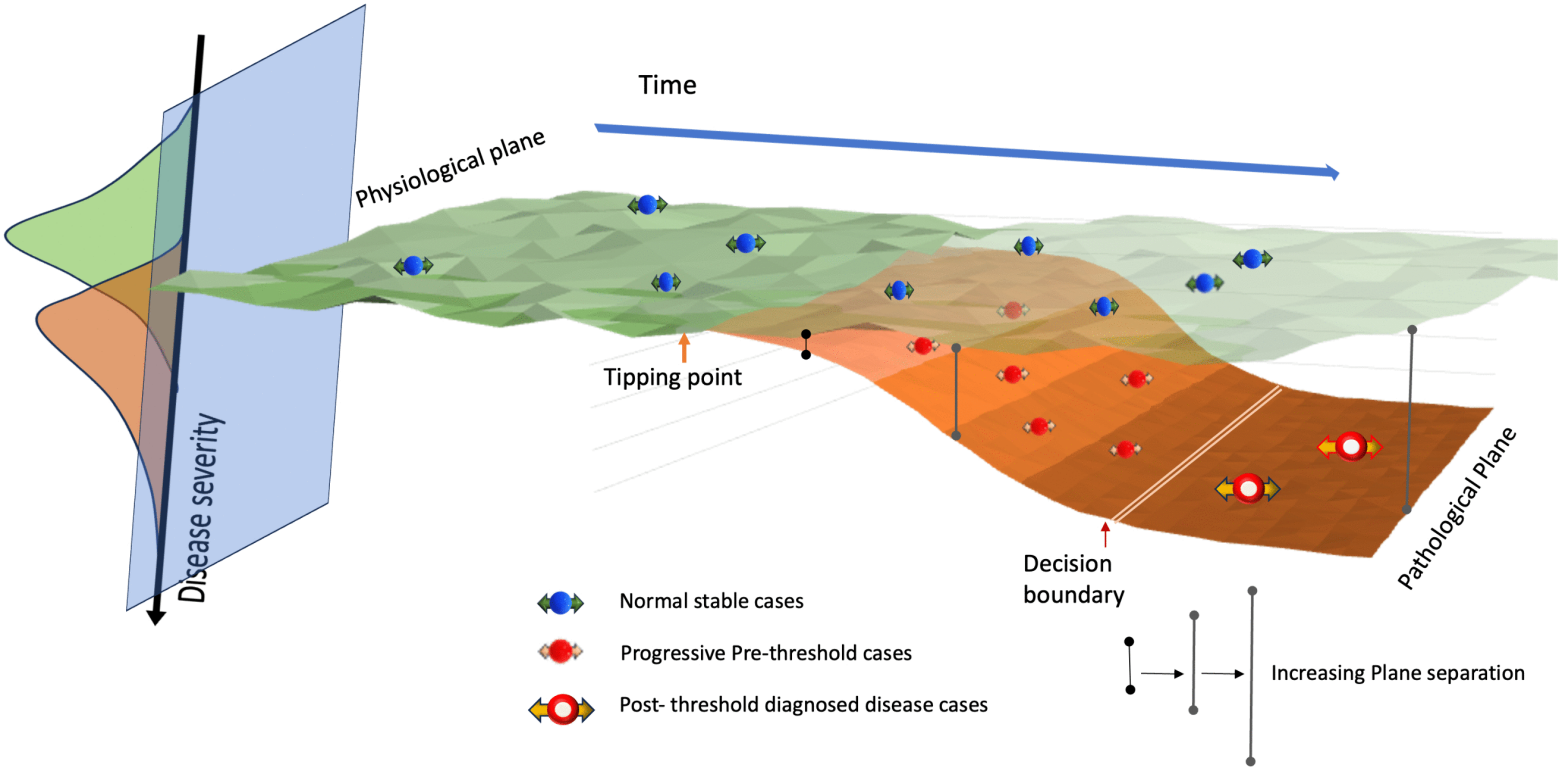
The two plane hypotheses and directional drift concept. Conceptual model of the two-plane hypothesis. Normal subjects remain within the physiological plane, while progressive cases drift onto the pathological plane after the tipping point.

We propose a two plane hypotheses and suggests that normal and disease states are better represented as existing on distinct geometric planes. The physiological plane is constrained by homeostasis. Values fluctuate but remain bounded and self-correcting. The disease plane has different behavior, what we term as ‘intentional directionality’ to differentiate from noise (which on measurement shows as non-intentional, non-directional movement). The tipping point marks the origin of the disease plane (Fig 3).

This transition is illustrated in the Fig 3, where normal subjects remain within the physiological plane with the passage of time, while progressively worsening pre-threshold and diagnosed cases drift on the pathological plane after the tipping event. The overlay of threshold-based distribution panel on the left of the image illustrates how classical models may produce overlapping decision regions, whereas the geometric model separates populations through angular divergence between planes. The exact moment of tipping may not be directly observable when intermittent sampling is being done compared to real-time tracking. In a clinical setting, this means that the tipping point may occur between follow-ups. However, the behavior before and after the tipping point can be traced back to a putative inflection point, giving mathematical support to this concept. Once tipping occurs, the subject may still appear within the normal range numerically. However, its new trajectory begins to follow the rules of the disease plane (Fig 3). The parameters continue to change and eventually cross conventional diagnostic thresholds, progressing further into disease.

The concept of the two planes can be extended beyond qualitative or illustrative value by mathematically defining them. This can help in creating a framework that documents the changes occurring on these planes around the tipping point. This quantification may help into extending it into clinical implications.

In the next section, we present a vector-based geometric framework that captures this transition in feature space.

## Methods

For this exploratory study, we first developed a geometric framework in which normal and disease planes, and their derived vector products, are defined. We then formalized the rules governing their interactions and applied this framework to a simulated dataset built on biological behavior described in the literature. The following is a narrative summary of the framework; complete mathematical derivations and a detailed commentary are provided in S1 Appendix.

### A. Framework Design

#### 1. Variable selection and feature space definition.

Two biologically meaningful variables, x and y, are selected to define the feature space. To ensure clinical interpretability and framework generalizability, selected variables should satisfy the following criteria: (i) established clinical relevance to the disease process; (ii) a biologically plausible directional relationship with disease progression; (iii) wide availability and routine use in clinical practice; (iv) preference for simple, direct measurements or low-complexity derived values; and (v) low inter-variable correlation in healthy subjects. In the current study, steepest keratometry (Kmax, diopters) and thinnest corneal thickness (TCT, microns) were selected as x and y respectively, based on their established roles in keratoconus progression [63,64].

#### 2. Normalization, scaling, and plane construction.

Variables are range-normalised using the physiological (normal) population as the reference. For a variable whose value *increases* with disease progression, the normalized value is:


$$\begin{array}{c} m^{\left(n\right)}=\frac{m-m_{min}}{R_{phy}} \end{array}$$ (1)

where *m* is the raw value, *m_min* is the minimum value in the physiological population, and $R_{phy}=\;m_{max}-m_{min}$ is the physiological range [Eq 2a, Supplementary S1 Appendix]. For variables that *decrease* with disease progression, the symmetric transformation applies [Eq. 2c, S1 Appendix]. This directional alignment ensures that worsening always maps in the positive direction for both variables, regardless of their raw units.

Normalized physiological subjects define a bounded reference plane $P_{phys}=[\text{0},\text{1}]\;\times [\text{0},\text{1}]$ (Eq 3a, Supplementary S1 Appendix). Disease subjects are scaled using the same physiological range (Eqs. 4a–4b, Supplementary S1 Appendix), placing them in a comparable but unbounded pathological plane $P_{path}=\left[\text{0},\varepsilon_{x}\right]\;\times \left[\text{0},\varepsilon_{y}\right]$, where $\varepsilon_{x},\;\varepsilon_{y}$ represent the scaled end-state disease maxima. Since this study focuses on early drift near the physiological boundary, end-state values serve only to define the upper limit of the pathological plane and are not modelled directly. Full derivations of both planes are provided in Supplementary S1 Appendix (Eqs. 2–5).

#### 3. Physiological noise scalar (η).

Physiological and observational variability are pooled into a single noise scalar η, derived from the within-subject standard deviation (Sw) of repeated measurements in the stable normal group. The final noise magnitude is defined as:


$$\begin{array}{c} \eta =\text{2}.\text{77}\cdot \sqrt{\frac{\text{1}}{q_{j}-\text{1}}\sum_{i=\text{1}}^{q_{j}}{\left({Sw_{j}}^{\left(x^{\left(n\right)},\;y^{\left(n\right)}\right)}\right)}^{\text{2}}\;} \end{array}$$ (2)

where ${Sw_{j}}^{\left(x^{\left(n\right)},\;y^{\left(n\right)}\right)}\;$is the joint within-subject standard deviation for physiological subject *j*, and the factor 2.77 corresponds to the 95% coefficient of repeatability (CR = $\text{1}.\text{96}\cdot \sqrt{\text{2}}\cdot \;Sw$) [65]. This extends the univariate CR concept (used previously by us for statistical threshold estimation in keratoconus progression) into a radial 95% confidence boundary in the joint feature space, serving as the denominator in the signal-to-noise framework described below [66]. The full per-subject Sw derivation, including the covariance term accounting for correlation between x and y, is provided in Supplementary S1 Appendix (Eqs. 6 a-f).

#### 4. Age-related physiological reference drift ($\vec{A}$).

Many physiological parameters change gradually with age, and this background drift must be distinguished from pathological change [67–69]. A linear age-related drift vector $\vec{A}$ ${=(x}_{t}^{\left(a\right)}$, $y_{t}^{\left(a\right)}$) was incorporated as a correction term in all subsequent vector calculations. Over the 2.5-year observation window of this exploratory study, age-related drift was assumed to be negligible; the correction terms were retained as placeholders for future iterations with longer follow-up. The full formulation is provided in Supplementary S1 Appendix (Eqs. 6g-j).

#### 5. Canonical disease vector ($\vec{D}$).

The direction of disease progression is captured as a pooled disease vector D, derived from the mean baseline-to-follow-up change across all confirmed progressive disease subjects (ED_P group), adjusted for age-related drift. For each pathological subject k, the age-adjusted change at each follow-up visit is computed and then averaged across visits and subjects to yield a single canonical direction vector $\vec{D}=(\overset{―}{{\Delta x}^{\left(d\right)}},\;\overset{―}{{\Delta y}^{\left(d\right)})}\;$ in the normalised feature space. This vector serves as the reference direction against which individual subject drift is compared. The complete derivation, including per-subject trajectory vectors and the pooling procedure, is provided in Supplementary S1 Appendix (Eqs. 6j–6q).

#### 6. Subject-specific drift vector $\vec{S_{u}}$.

For any subject under evaluation (denoted u), the drift vector $\vec{S_{u}}\;$is computed as the age-adjusted change from baseline to follow-up visit t, using the same scaling principles applied to pathological subjects. The magnitude $\left|\vec{S_{u}}\right|$quantifies how far the subject has moved in the feature space, and the angle $\theta_{\vec{\text{u}}}\;$defines the direction of that movement. Full expressions for $\vec{S_{u}}$, $\left|\vec{S_{u}}\right|,$and $\theta_{\vec{\text{u}}}$are provided in Supplementary S1 Appendix (Eqs. 6r–6t).

#### 7. Derived metrics: MNR, DEM, and Composite Drift Score (CDS).

**Magnitude-to-Noise Ratio (MNR).** A subject's drift is meaningful only if it exceeds the expected physiological noise. Unit-dependent absolute change thresholds are poorly generalisable across variables and clinical domains. We therefore define a dimensionless signal-to-noise ratio:


$$\begin{array}{c} MNR=\frac{\left|\vec{{\text{S}}_{u}}\right|}{\eta } \end{array}$$ (3)

An MNR > 1 indicates that the subject's displacement exceeds the 95% physiological noise boundary. Just as a radio-based system uses SNR to deduce a meaningful signal over background static, the MNR intends to deduce subject-level change over the expected noise.

**Directional Emphasis Multiplier (DEM).** Drift magnitude alone is insufficient, a subject moving perpendicular or opposite to the disease direction should not be flagged. The cosine of the angle between the subject vector $\vec{S_{u}}$ and the canonical disease vector $\vec{D}\;$ measures angular alignment (cos $\emptyset_{u}$= 1 for perfect alignment, 0 for orthogonal, −1 for opposite). To reward strong alignment quadratically and penalise misalignment, we apply a signed cosine-square weighting:


$$\begin{array}{c} {DEM}_{u}=\text{ cos}\left(\emptyset_{u}\right).\left|\text{cos}\emptyset_{u}\right| \end{array}$$ (4)

DEM ranges from −1 to +1. Movement precisely aligned with the disease vector yields DEM = 1; movement in the opposite direction yields DEM = −1 (which may prove useful as a treatment-response indicator in future work). The signed-square function is customizable and steeper alternatives such as cos³(ϕ) can be used when tighter directional specificity is required.

**Composite Drift Score (CDS).** The final metric combines both directional and magnitude components into a single, unitless score:


$$\begin{array}{c} {CDS}_{u}={DEM}_{u}\cdot {MNR}_{u} \end{array}$$ (5)

This scalar, unitless value reflects both the strength and directionality of pathological drift and forms the basis for early detection in this framework. It is intuitively set at a threshold of ≥1.0 (perfect alignment with disease process and change greater than seen with noise, which can vary over multiple case scenarios as we discuss later in results and discussion).

#### 8. Extension to higher dimensions and correlated variables (Mahalanobis approach).

The Euclidean CR-based noise scalar η described above performs well for the 2D, low-covariance setting of this study (Kmax and TCT show minimal correlation in normal eyes). However, for datasets with three or more variables, or where inter-variable covariance is substantial, the Euclidean approach may underestimate the true noise boundary. Mahalanobis distance has been noted to counter this issue [70–72]. Therefore, we derived a Mahalanobis distance (MD)-based alternative noise metric, yielding MNR(MD) and CDS(MD) as covariance-adjusted analogues of MNR and CDS:


$$\begin{array}{c} {MNR}_{u}^{\left(MD\right)}=\;\frac{{MD}_{u,t}}{\text{2}.\text{4}\text{5}}\; \end{array}$$ (6)

$$\begin{array}{c} {CDS}_{u}^{\left(MD\right)}={DEM}_{u}\cdot {MNR}_{u}^{\left(MD\right)} \end{array}$$ (7)

The divisor 2.45 corresponds to the square root of the 95th percentile of the chi-square distribution with 2 degrees of freedom (√5.99 ≈ 2.45), providing a multivariate equivalent of the univariate CR threshold. In our synthetic dataset, both CR-based and MD-based metrics showed consistent directional trends across groups, with MD-based scores yielding numerically higher values in progressive cohorts. For this initial 2D study, we retain the CR-based MNR and CDS as primary metrics for their clinical interpretability; the MD framework is reserved for future higher-dimensional implementations. The complete Mahalanobis derivation, including covariance matrix construction and the chi-square reference boundary, is provided in Supplementary S1 Appendix (Eqs. 8a–8i).

**B. Application to synthetic subjects**: With the above framework established, we applied the above steps 1–8 on a labeled synthetic dataset composed of four distinct subject groups (n = 1000 per group), representing a different disease stage or behavioral phenotype. For this exploratory study, we wanted to explore a disease with domain knowledge before expanding to others in future studies, so that we are aware of the expected physiological and pathological behaviors. Therefore, we modeled normal cornea and early keratoconus for this study and the two variables used were steepest Keratometry (Kmax in diopters) and thinnest corneal thickness (TCT) in microns. The synthetic data was generated using normal and abnormal ranges, rate of progression, and intra-measurement standard deviation provided in the literature [63,64]. The complete data generation methodology, including randomization logic, Gaussian noise parameters, seed values, and parameter distributions, is provided in S2 Appendix. The excel sheet with the final data is provided as S3 Appendix and is available publicly at https://doi.org/10.17605/OSF.IO/2JNQF (CC-BY 4.0 International). An illustrative pdf with the framework of the construction is supplied as S4 Appendix. To prevent any future chances of data redundancy or overfitting, we did not use any of our previous studies or our published data with normative corneal or keratoconus data.

To prevent any future chances of data redundancy or overfitting, we did not use any of our previous studies or our published data with normative corneal or keratoconus data.

1. **The 4 subject groups were:**

a. *Stable normal cases:* Used to define the reference range for physiological noise η  and physiological reference drift$\;\vec{R}$.
b. *Stable (early) disease cases*: served as anchor points for comparison with progressive disease. These cases belong clearly to the pathological plane (no suspects or borderline case) without directional progression.
c. *Progressive (early) disease cases*: These subjects also belonged to the pathological plane and have with similar baselines as (b). They were simulated to reflect realistic but stochastic disease progression. Changes in the individual variables x,y were adjusted to trend over worsening values consistent with known early disease behavior over time. The disease vector $\vec{D}$ was calculated from this group.
d. Progressive normal-range cases: This group represents the primary target of our framework: subjects whose baseline values fell within traditional “normal” ranges (based on Gaussian cutoffs or consensus thresholds), but whose homeostatic compensatory mechanisms have failed, leading them to drift beyond the biological tipping point and thus, by definition, into the pathological plane. Variables x,y were simulated to reflect stochastic but directionally worsening trends, consistent with known early disease behavior over time. To maintain biological plausibility, the average rate of change in this group was scaled to approximately 80% of that used for the early progressive disease group (Group C). This conservative modeling assumption reflects the current lack of direct empirical data on progression dynamics in this pre-diagnostic window. The drift parameters used here are customizable and can be refined in future models using more granular longitudinal datasets.

2) Computations in the synthetic dataset: noise (η) and disease vector ($\vec{\text{ D})}$ were computed at the dataset level, and $\vec{\text{ U}},\text{ cos}(\phi )$, DEM, MNR, and CDS were computed at the subject level.

## Results

**A. Group Characteristics and Internal Validation:** The synthetic exploratory dataset included four simulated groups (n = 1000 each): Normal Stable (Norm), Early Disease Stable (ED_S), Early Disease Progressive (ED_P), and Pre-threshold Progressive (PT_P).

As noted previously, in this study, the two variables x and y were assigned values from Kmax and TCT, respectively, such that Kmax → x and TCT → y. All subsequent analyses refer to these generalized variables x and y for clarity and broader applicability.

Group means and standard deviations for baseline (t0) and final follow-up (t5) values of (x,y) and normalized variables ${\;x}^{\left(n\right)},\;y^{\left(n\right)}\;$confirmed expected characteristics hypothesized while modeling the framework. Both the groups designed to worsen with time (ED_P, PT_P) showed worsening of variables with time, while the groups designed to remain stable (Norm, ED_S) remained relatively unchanged (Tables 1 and 2).

**Table 1:** Raw Variables (x,y) at visits t0 to t5.

		Early Disease Progressive		Early Disease Stable		Normal Stable		Pre_Threshold Progressive		Total	
Raw variable		Mean	Std. Deviation	Mean	Std. Deviation	Mean	Std. Deviation	Mean	Std. Deviation	Mean	Std. Deviation
x	t0	48.72	2.39	48.75	2.37	43.70	0.99	43.70	0.99	46.22	3.11
	t1	49.09	2.42	48.75	2.37	43.70	1.02	44.01	1.01	46.39	3.14
	t2	49.47	2.43	48.74	2.36	43.70	1.01	44.33	1.04	46.56	3.16
	t3	49.84	2.45	48.75	2.38	43.70	1.01	44.65	1.06	46.73	3.20
	t4	50.22	2.45	48.75	2.38	43.69	1.02	44.97	1.08	46.91	3.25
	t5	50.59	2.46	48.75	2.37	43.71	1.01	45.29	1.09	47.08	3.30
y	t0	499.84	26.41	498.26	26.27	544.15	18.60	543.39	17.96	521.41	31.84
	t1	493.55	26.78	498.25	26.64	544.18	18.96	538.82	18.37	518.70	32.51
	t2	486.99	27.21	498.11	26.64	544.09	18.87	534.23	18.81	515.85	33.32
	t3	480.45	27.60	498.05	26.70	544.14	18.91	529.67	19.11	513.08	34.38
	t4	473.73	28.36	498.24	26.67	543.96	19.00	524.99	19.46	510.23	35.66
	t5	467.08	28.87	498.13	26.79	544.16	18.97	520.64	20.02	507.50	37.25

**Table 2:** Directionality and range normalized variables (x^(n,)^ y^(n)^) at visits t0 to t5.

		Early Disease Progressive		Early Disease Stable		Normal Stable		Pre_Threshold Progressive		Total	
Normalized variable	Visit	Mean	Std. Deviation	Mean	Std. Deviation	Mean	Std. Deviation	Mean	Std. Deviation	Mean	Std. Deviation
x^(n)^	t0	1.46	0.46	1.46	0.45	0.50	0.19	0.50	0.19	0.98	0.59
	t1	1.53	0.46	1.46	0.45	0.50	0.19	0.56	0.19	1.01	0.60
	t2	1.60	0.46	1.46	0.45	0.50	0.19	0.62	0.20	1.05	0.60
	t3	1.67	0.47	1.46	0.45	0.50	0.19	0.68	0.20	1.08	0.61
	t4	1.74	0.47	1.46	0.45	0.50	0.19	0.74	0.21	1.11	0.62
	t5	1.81	0.47	1.46	0.45	0.50	0.19	0.80	0.21	1.15	0.63
y^(n)^	t0	0.95	0.26	0.97	0.26	0.51	0.18	0.52	0.18	0.74	0.32
	t1	1.01	0.27	0.97	0.26	0.51	0.19	0.57	0.18	0.77	0.32
	t2	1.08	0.27	0.97	0.26	0.51	0.19	0.61	0.19	0.79	0.33
	t3	1.14	0.27	0.97	0.26	0.51	0.19	0.66	0.19	0.82	0.34
	t4	1.21	0.28	0.97	0.26	0.52	0.19	0.70	0.19	0.85	0.35
	t5	1.28	0.29	0.97	0.27	0.51	0.19	0.75	0.20	0.88	0.37

**Fig 4:**
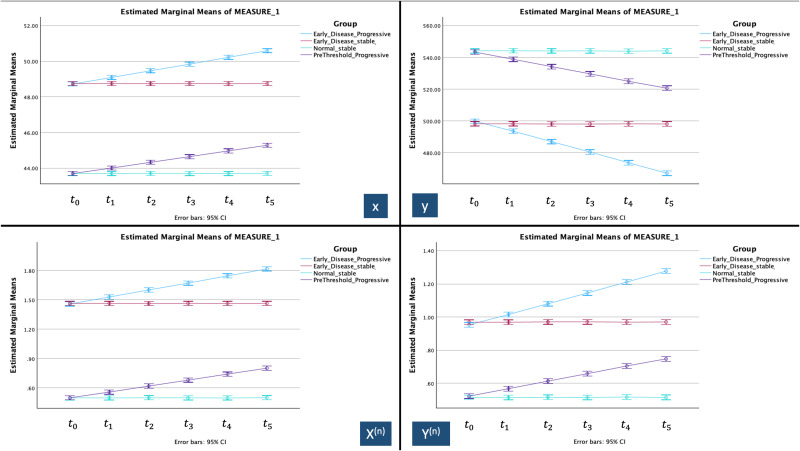
Estimated marginal means of x and y $x^{\left(n\right)}$ values normalized(top row) and their corresponding and $y^{\left(n\right)}$ (bottom row) across all 6 timepoints (t0- t5) for all four cohorts.

A repeated-measures ANOVA on raw x,y values across time (t0−t5) revealed a statistically significant main effect of time (p < .001), and of time × group interaction for all comparison (p < .001). Plotting the raw values demonstrated diverging trajectories in progressive groups (increasing x, decreasing y) while stable and normal groups remained flat. Normalized values ($x^{\left(n\right)},\;y^{\left(n\right)}$confirmed that progression occurs along a biologically plausible axis and further highlight the temporal separation between disease and stable behavior (Fig 4).

This ensured that the synthetic dataset followed the planned intention of creating 4 different datasets. The randomization and simulation did result in the trends we wanted to mimic, reassuring internal consistency.

**B: Indices’ performance** Next, we wanted to evaluate the performance of the indices we had conceptualized. The progressive groups showed a significant increase in the three metrics DEM, MNR, and CDS, whereas the stable groups did not show a significant change (Table 3). Both the sets of CR derived and the Mahalanobis distance (MD) derived metrics (MNR, MNR^(MD),^ CDS, CDS^(MD)^) showed this trend. MNR^(MD)^ and CDS^(MD)^ both showed higher values for the progressives group.

**Table 3:** Change in the derived indices over follow-ups (t1 → t5) compared to baseline (t0).

		Early Disease Progressive		Early Disease Stable		Normal Stable		Pre-Threshold Progressive		Total	
		Mean	SD	Mean	SD	Mean	SD	Mean	SD	Mean	SD
**DEM — Directional Emphasis Multiplier**											
**DEM_CR**	*t1-t0*	0.79	0.26	0.01	0.61	−0.01	0.62	0.72	0.31	0.38	0.61
	*t2-t0*	0.89	0.15	0.01	0.63	0.01	0.62	0.85	0.20	0.44	0.63
	*t3-t0*	0.93	0.11	0.03	0.62	0.00	0.61	0.90	0.15	0.46	0.63
	*t4-t0*	0.95	0.08	0.00	0.62	0.00	0.62	0.92	0.11	0.47	0.64
	*t5-t0*	0.98	0.04	0.02	0.71	0.03	0.71	0.97	0.05	0.50	0.69
**DEM_MD**	*t1-t0*	0.79	0.26	0.01	0.61	−0.01	0.62	0.72	0.31	0.38	0.61
	*t2-t0*	0.89	0.15	0.01	0.63	0.01	0.62	0.85	0.20	0.44	0.63
	*t3-t0*	0.93	0.11	0.03	0.62	0.00	0.61	0.90	0.15	0.46	0.63
	*t4-t0*	0.95	0.08	0.00	0.62	0.00	0.62	0.92	0.11	0.47	0.64
	*t5-t0*	0.98	0.04	0.03	0.71	0.03	0.71	0.97	0.05	0.50	0.69
**MNR — Magnitude-to-Noise Ratio**											
**MNR_CR**	*t1-t0*	0.95	0.38	0.47	0.25	0.35	0.18	0.78	0.33	0.64	0.38
	*t2-t0*	1.82	0.55	0.46	0.24	0.35	0.18	1.46	0.51	1.02	0.75
	*t3-t0*	2.67	0.67	0.45	0.25	0.35	0.18	2.13	0.62	1.40	1.13
	*t4-t0*	3.56	0.79	0.47	0.25	0.35	0.19	2.83	0.73	1.80	1.52
	*t5-t0*	4.44	0.91	0.46	0.25	0.35	0.18	3.49	0.82	2.18	1.92
**MNR_MD**	*t1-t0*	1.07	0.42	0.52	0.27	0.39	0.21	0.87	0.37	0.71	0.32
	*t2-t0*	2.03	0.62	0.51	0.28	0.39	0.20	1.62	0.56	1.14	0.41
	*t3-t0*	2.99	0.75	0.52	0.28	0.39	0.21	2.37	0.69	1.57	0.48
	*t4-t0*	3.99	0.89	0.51	0.27	0.38	0.20	3.14	0.81	2.01	0.54
	*t5-t0*	4.97	1.02	0.65	0.35	0.48	0.25	3.88	0.90	2.49	0.63
**CDS — Composite Drift Score**											
**CDS_CR**	*t1-t0*	0.79	0.42	0.00	0.31	0.00	0.24	0.60	0.37	0.35	0.49
	*t2-t0*	1.64	0.60	0.00	0.32	0.01	0.24	1.27	0.55	0.73	0.87
	*t3-t0*	2.50	0.73	0.01	0.32	0.00	0.24	1.93	0.67	1.11	1.24
	*t4-t0*	3.39	0.84	0.00	0.33	0.00	0.25	2.62	0.76	1.50	1.64
	*t5-t0*	4.34	0.93	0.01	0.37	0.01	0.27	3.38	0.82	1.93	2.07
**CDS_MD**	*t1-t0*	0.88	0.47	0.00	0.36	−0.01	0.27	0.67	0.41	0.39	0.38
	*t2-t0*	1.84	0.67	0.00	0.01	0.01	0.28	1.41	0.61	0.81	0.39
	*t3-t0*	2.79	0.82	0.00	0.01	0.00	0.27	2.15	0.74	1.24	0.46
	*t4-t0*	3.79	0.94	0.00	0.01	0.01	0.27	2.91	0.85	1.68	0.52
	*t5-t0*	4.86	1.04	0.01	0.43	0.01	0.39	3.75	0.92	2.16	0.69

CR = Euclidean coefficient-of-repeatability based metric. MD = Mahalanobis distance-based metric. DEM_MD = DEM_CR (values identical: DEM is a purely angular measure, independent of the noise metric).

MNR(MD) significantly higher than MNR(CR) across all groups (paired t-test, all p < 0.001); mean differences: NS 0.13, ED_S 0.19, ED_P 0.53, PT_P 0.39. CDS difference non-significant in stable groups (NS p = 0.49; ED_S p = 0.95), consistent with near-zero DEM suppressing MNR differences at the CDS level (CDS = DEM × MNR).

Classification concordance at CDS ≥ 1.0: 100% for progressive groups (kappa = 1.0); 99.7% (NS) and 98.5% (ED_S) for stable groups. All values mean ± SD. DEM: Directional Emphasis Multiplier. MNR: Magnitude-to-Noise Ratio. CDS: Composite Drift Score.

To formally evaluate agreement between the two approaches, paired t-tests and classification concordance analysis were performed at t5. MNR(MD) was significantly higher than MNR(CR) across all groups (all p < 0.001), with mean differences of 0.13 in NS, 0.19 in ED_S, 0.53 in ED_P, and 0.39 in PT_P, consistent with the MD approach's greater sensitivity to multivariate deviation. However, this did not translate into a meaningful CDS difference in stable groups (NS: mean difference 0.005, p = 0.49; ED_S: mean difference 0.001, p = 0.95). This is consistent with the structure of the framework: CDS = DEM × MNR, where near-zero DEM values in stable subjects (NS mean DEM 0.032; ED_S mean DEM 0.025) suppress any MNR difference at the CDS level, regardless of which noise metric is used. In progressive groups where DEM approaches 1.0, the CDS difference between methods closely tracks the MNR difference (ED_P mean difference 0.52, PT_P mean difference 0.37; both p < 0.001), which is numerically larger but clinically consistent. Classification concordance at CDS ≥ 1.0 was 100% for both progressive groups (kappa = 1.0): every subject flagged by CR-based CDS was identically classified by MD-based CDS and vice versa. In stable groups, concordance was 99.7% (NS) and 98.5% (ED_S), with the small number of discordant cases (3 and 12 respectively) representing MD-only flags in subjects with sub-threshold CR-based values. These findings confirm that while MD-based metrics yield numerically higher scores, clinical classification is identical between the two approaches in this 2D, low-covariance setting, supporting the use of the simpler CR-based approach as the primary metric for this study.

For the purpose of this initial study, we continue to use CR-derived MNR and CDS for the rest of the evaluation and would reserve further exploration with MD derived metrics in a higher dimensional dataset in the future.

**Fig 5:**
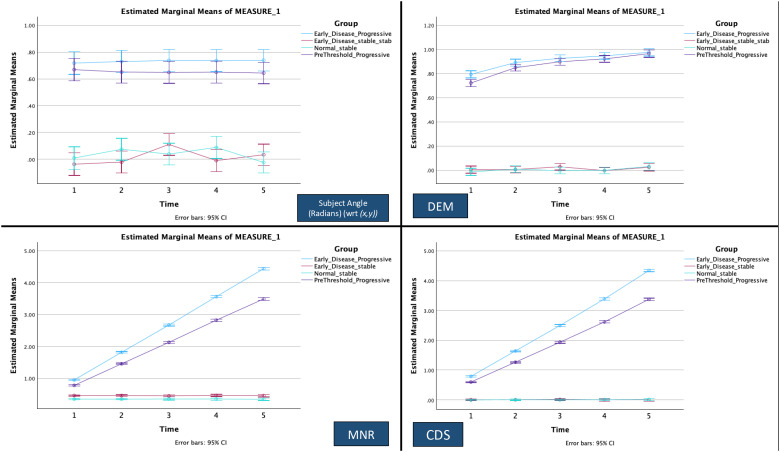
Estimated marginal means of four derived vector-based metrics over the five follow-ups (t1−t5)  grouped by cohort: Top-left: Subject vector angle (in radians) with respect to the x,y coordinate system. Top-right: Directional Emphasis Multiplier (DEM). Bottom-left: Magnitude-to-Noise Ratio (MNR). Bottom-right: Composite Drift Score (CDS).

As shown in Fig 5, the subject vector angle (top-left panel) remained stable over time, with no significant time × group interaction, reflecting that subject maintained a consistent drift direction in the x,y space (Repeated-measures ANOVA p > 0.5).

In contrast, Directional Emphasis Multiplier (DEM) increased significantly over time in progressive groups (repeated-measures ANOVA, p < 0.001) (top-right panel). This may reflect improving alignment with the disease trajectory and not a large change in direction. This could also suggest the self-correcting nature of the index due to repeated measures from the baseline over follow-up. Again, as this was synthetic data, we do expect more noisiness in real cases and therefore this finding if repeated in those groups, needs to be explored further. Also, with time the pre-threshold progressive cases (PT_P) trended to match the performance of the early progressive disease (ED_P). This again is suggestive of a possible stronger alignment with confirmed disease process with time. This was not a planned or hard coded step but is explainable on disease behavior, further suggesting that even though this data was synthetic, did show patterns we expected.

The Magnitude-to-Noise Ratio (MNR) (bottom-left panel) rose in progressive groups, indicating that observed drift exceeded physiological noise boundaries (repeated-measures ANOVA, p < 0.001).

Finally, the Composite Drift Score (CDS) (bottom-right panel), showed a rise in Early Disease Progressive and Pre-threshold Progressive groups, while remaining flat in stable cohorts (repeated-measures ANOVA, p < 0.001).

Taken together, these vector-based metrics demonstrated strong discriminative behavior across simulated disease states, justifying the potential for further exploration for trajectory analysis and diagnostic comparison in more complicated or real-life datasets.

**Fig 6:**
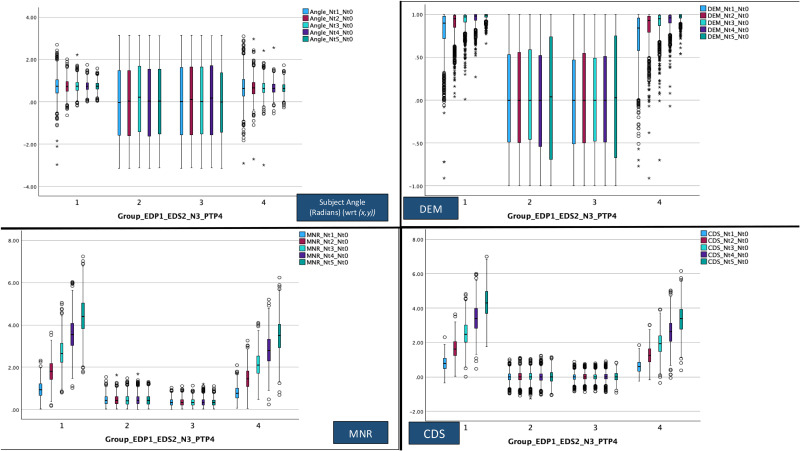
Grouped boxplots of four derived metrics over time (t1 to t5, relative to baseline t0) Top-left: Subject vector angle (in radians) with respect to the  x,y  coordinate system. Top-right: Directional Emphasis Multiplier (DEM). Bottom-left: Magnitude-to-Noise Ratio (MNR). Bottom-right: Composite Drift Score (CDS).

To visualize how directional drift evolves over time and contributes to the Composite Drift Score, we constructed a four-panel boxplot montage (Fig 6).

Subject angle (top-left panel) revealed that progressive groups converged directionally toward the canonical disease vector, while stable and normal groups remained without a clear directional trend. This angular trend was reflected in rising DEM values (top-right panel), which translated into increased alignment. Combined with the rising MNR (bottom-left panel), CDS values (bottom-right panel), rose in progressive cohorts ED_P and PT_P, confirming that directional alignment and supra-noise drift both contributed to the CDS scores getting higher.

**Fig 7:**
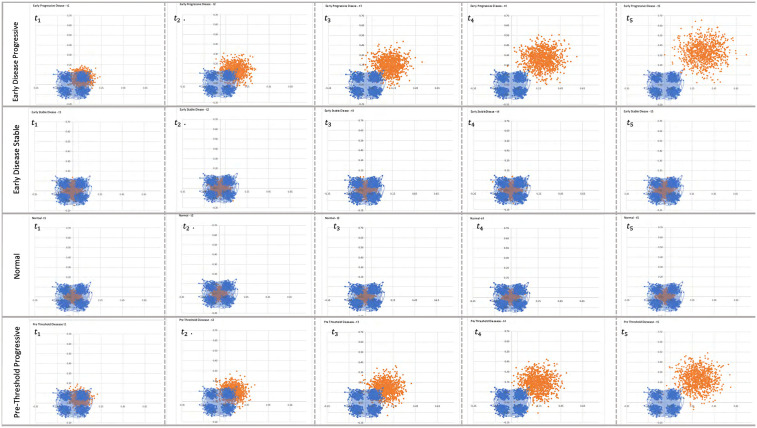
Overlay scatter plots showing subject-level displacement vectors (orange) over baseline noise cloud (blue), across cohorts and timepoints.

**C. Subject-level stochasticity visualization:** Even though we induced Gaussian noise in our samples as described in the methods, at the level of measures of central tendency it is difficult to see if the outcomes created were artificially smooth without. To assess intra-individual variability visually, we mapped each subject's vector displacement over time. Fig 7 shows directional scatter plots for all four groups across five follow-up time points, with simulated subject vectors (orange) overlaid on the baseline physiological noise cloud (blue).

The stable and normal cohorts remained within the noise cloud over the follow-ups. The progressive groups showed clear but directional trends away from the noise cloud, which varied in speed between subjects. This further gave visual support to the drift varying in quantity but similar in the direction in the subjects where it was intended.

Perhaps, we should discuss the method of noise cloud representation in this visual analogy in more detail: As CR is a positive number, plotting the CR only would have artificially resulted in a data set limited to quadrant I of the cartesian system. In constructing the baseline physiological appearing noise cloud, we aligned the noise distribution with the biological directionality of change observed at the last visit (i=5)


$$\begin{array}{c} \left[x_{j}^{\left(noise\right)},\;y_{j}^{\left(noise\right)}\right]=\;\left(\text{2}.\text{7}\text{7}\frac{\left|{\delta x}_{j,i}^{\left(n\right)}\right|}{{\delta x}_{j,i}^{\left(n\right)}}{Sw}_{j}^{\left(x^{\left(n\right)}\right)}\right),\;\left(\text{2}.\text{7}\text{7}\frac{\left|{\delta y}_{j,i}^{\left(n\right)}\right|}{{\delta y}_{j,i}^{\left(n\right)}}{Sw}_{j}^{\left(y^{\left(n\right)}\right)}\right) \end{array}$$ (8)

Where the${\;x}_{j}^{\left(noise\right)},\;y_{j}^{\left(noise\right)}$are the coordinates of the noise cloud for subject *j*, $\left|{\delta x}_{j}^{\left(n\right)}\right|,\left|{\delta y}_{j}^{\left(n\right)}\right|$ are the absolute values of ${\;\delta x}_{j}^{\left(n\right)}$,${\delta y}_{j}^{\left(n\right)}$, the change in the normalized x and y variables at the last follow-up i=5, and ${{Sw}_{j}^{\left(x^{\left(n\right)}\right)},\;Sw}_{j}^{\left(y^{\left(n\right)}\right)}$ are subject level intra-measurement standard deviations which when multiplied with 2.77 gives the CR for that subject. Though this approximation is not mathematically equivalent to the CR we used as a pooled value, hopefully, it gives a visual representation of the noise cloud primarily at an intuitional level. This could also be useful as we further deal with more stochastic and subject-level data in future work.

**Fig 8:**
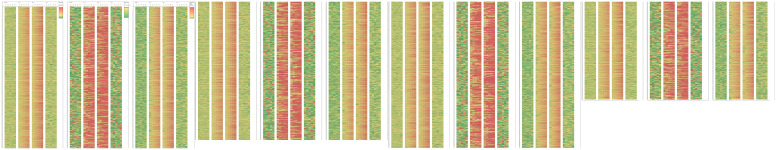
Heatmap visualization of subject-level trajectories for all the cases from each group for CDS, Cosine angle, and Subject vector magnitude across five follow-up timepoints. Rows represent individual subjects; warmer colors indicate increasing values.

Also, Fig 8 presents heat maps of subject-level evolution for all the follow-ups of all the cases for CDS, the cosine angle between disease and subject vectors, and magnitude of subject vector.

Progressive cohorts show increasing intensity (green to orange to red) in both alignment and magnitude, consistent with disease-like drift accumulation. In contrast, stable disease and normal groups show lesser changes in the hues from the cooler colors over follow-ups. Together, these visualizations help to demonstrate the biological plausibility and internal variability of the synthetic dataset, and similar methods could be used in future work to compare the internal variability of more complicated datasets.

D. Sampling Frequency Sensitivity Analysis.

As a trajectory-based framework, CDS depends on the availability of serial measurements: fewer follow-up visits mean fewer opportunities to detect drift before conventional thresholds are crossed. To quantify this expected relationship, four clinically relevant sampling cadences were modelled in the PT_P group (n = 1000): 6-, 12-, 18-, and 24-month intervals, using the visits available within the 2.5-year observation window. The detection criterion was CDS ≥ 1.0 at any available visit. Lead time was calculated as the interval between first CDS flag and conventional TCT threshold crossing (TCT ≤ 500 µm, univariate threshold crossing as an example) in the 156 PT_P subjects who crossed the threshold during the observation period. Results are summarized in Table 4.

**Table 4:** Effect of follow-up cadence on CDS detection sensitivity and lead time in the Pre-Threshold Progressive group (n = 1000).

Follow-up cadence	Visits available	Detection rate	Mean first detection	Mean lead time before threshold*
**6-month**	t1, t2, t3, t4, t5	99.8% (998/1000)	13.4 months	11.3 months (SD 6.9)
**12-month**	t2, t4	98.5% (985/1000)	15.7 months	10.1 months (SD 6.7)
**18-month**	t3	92.7% (927/1000)	18.0 months	7.5 months (SD 4.6)
**24-month**	t4	98.5% (985/1000)	24.0 months	3.5 months (SD 3.0)

*Lead time = interval between first CDS ≥ 1.0 flag and conventional TCT threshold crossing (TCT ≤ 500 µm); n = 156 PT_P subjects crossing threshold during observation window. Each timepoint represents 6 months from baseline (t1 = 6m, t2 = 12m, t3 = 18m, t4 = 24m, t5 = 30m).

Detection rate = proportion of PT_P subjects (n = 1000) with CDS ≥ 1.0 at any available visit within the 2.5-year observation window. At 24-month cadence, lead time compresses to 3.5 months despite maintained detection rate, as most detections occur close to or at the time of threshold crossing. CDS: Composite Drift Score. TCT: Thinnest Corneal Thickness. PT_P: Pre-threshold Progressive group.

As expected, detection rate and lead time both declined as sampling frequency decreased. With 6-month follow-up, 99.8% of PT_P subjects were flagged at a mean of 13.4 months, with a mean lead time of 11.3 months ahead of threshold crossing. At 12-month intervals, detection rate was maintained at 98.5% with mean lead time of 10.1 months, a modest reduction consistent with the loss of alternate visits. At 18-month intervals, detection fell to 92.7% and lead time to 7.5 months. At 24-month intervals, detection recovered to 98.5% as the t4 visit captures most progressors by that point, but lead time compressed to 3.5 months, the shortest across all cadences and leaving little clinical window for intervention before threshold crossing.

These results are an expected property of any trajectory-based detection system and are presented here to quantify the trade-off rather than as a novel finding. In this dataset and for the disease model used, the framework's pre-threshold advantage is preserved under annual review and diminishes substantially at intervals beyond 18 months. The dependence on sampling frequency is acknowledged as a limitation of the framework and is discussed accordingly. Future work will explore cadence-specific optimization for individual disease domains and progression rate profiles.

## Discussion

This novel framework quantifies directional progression instead of relying solely on value-based thresholds. In this paper, we have conceptualized and then evaluated a geometric framework rooted in biological plausibility and clinical interpretability. The framework is derived from previous work in the fields of medicine, biomedical imaging, and signal processing. It derives inspiration from concepts in ecology, systems biology, and physics [56–59]. This is also an extension from our previous studies of univariate & bivariate range normalization, rate of change assessment and predictive modeling in keratoconus, use of repeatability indices for clinical decision-making, and modeling the role of directional alignment in the disease process [66,73,74]. The goal of this paper is to formalize a clinical intuition many physicians have when population-based cutoffs, which have historically worked well, lag in predicting patient’s unique changes [60–62]. We should also discuss the use of synthetic data in the study. Similar approaches using synthetic or semi-synthetic disease progression models have been used previously [75–79]. There are relatively large sizes of keratoconus, Fuchs dystrophy, and normal eye databases, and we have also reported findings from our databases. However, most of these are cross-sectional [80–83]. Subjects who are clinically normal are often not followed up serially in a systemic fashion. Therefore, we decided to use a more customizable method such as an Excel based dataset generation vs publicly available or pre-generated cross sectional synthetic datasets in this initial explorative study.

Often, correlated biomarkers lead to overfitting and interpretation difficulty [84, 85]. Therefore, the parsimonious design of this model is an attempt to safeguard against overfitting. While there can be exceptions, the initial plan should be to include 2 or 3 parameters that are used for threshold-based screening. The reason to use parameters already being used commonly in threshold-based testing is to keep the clinical interpretability intact due to preexisting familiarity in clinicians for those parameters (directionality and not necessarily the numerical thresholds). Continuing from our example of keratoconus, studies have shown the advantage of corneal wavefront measurement as a more sensitive marker for keratoconus diagnosis, and Brillouin microscopy can detect keratoconus much earlier than conventional testing **[86]**. However, most ophthalmologists will know that the cornea gets thinner and steeper with keratoconus and will be able to relate to keratometry and pachymetry at a more intuitive level than corneal wavefront [4,28].

As an expansion of scope, we visualize this system to be a framework that, if proven, may be transferable to the more comprehensive practice physicians such as a primary care physician or a comprehensive ophthalmologist who can work along with subspecialists. Therefore, the common interpretability of the parameters becomes important.

The use of relatively more esoteric parameters and complex indices has an undeniable role in severity mapping for advanced diseases, but the current framework is aimed to supplement that by working in a hitherto unexplored area **[**3,5,74**].** Our geometric vector-plane framework complements prior approaches that use information technologies to process multidimensional physiological state-spaces for health monitoring and intelligent decision support [87].

It is worth discussing the place of the CDS framework within the landscape of existing longitudinal monitoring approaches. Reference change values (RCV), derived from within-subject biological variation and analytical imprecision, are the most widely used method for flagging clinically significant interval change in a single variable. [31] CDS extends this concept in two important directions: it operates simultaneously across multiple variables in a shared feature space, and it incorporates directional alignment with a disease-specific trajectory rather than treating any sufficiently large change as meaningful regardless of its direction.

Composite multiparameter indices such as the Belin-Ambrósio Enhanced Ectasia Display in keratoconus, the AGIS score in glaucoma, or KDIGO staging in chronic kidney disease offer powerful classification but are designed to characterize disease severity or cumulative damage rather than to detect directional pre-threshold drift in subjects still classified as normal [88–90]. Machine learning classifiers for early disease detection can achieve high discriminative accuracy in well-powered datasets but typically require large, labelled training cohorts, offer limited interpretability at the individual subject level, and are not inherently designed around the concept of directional biological drift [91, 92]. CDS is not intended to replace any of these approaches. Rather, it is designed to operate upstream of them in the pre-threshold longitudinal window where a subject's measurements remain numerically normal, yet their trajectory has already diverged from physiological noise. In this sense, CDS is less a competitor to existing diagnostic tools and more a temporally earlier layer of the same clinical decision process.

However, one may be concerned if this is an oversimplification (using lower order, linear approximations rather than the complex black box style models). Clinicians and researchers are aware of the complex stochastic process of disease progression after the clinical thresholds are triggered up to complete loss of structure or function or both (as the case may be) [93**–**95**].** Simply put, disease progression starts and stops, accelerates, and decelerates, and multiple genetic and environmental roles need to be modeled when mapping the entire disease process **[**96, 97**].**

**Fig 9:**
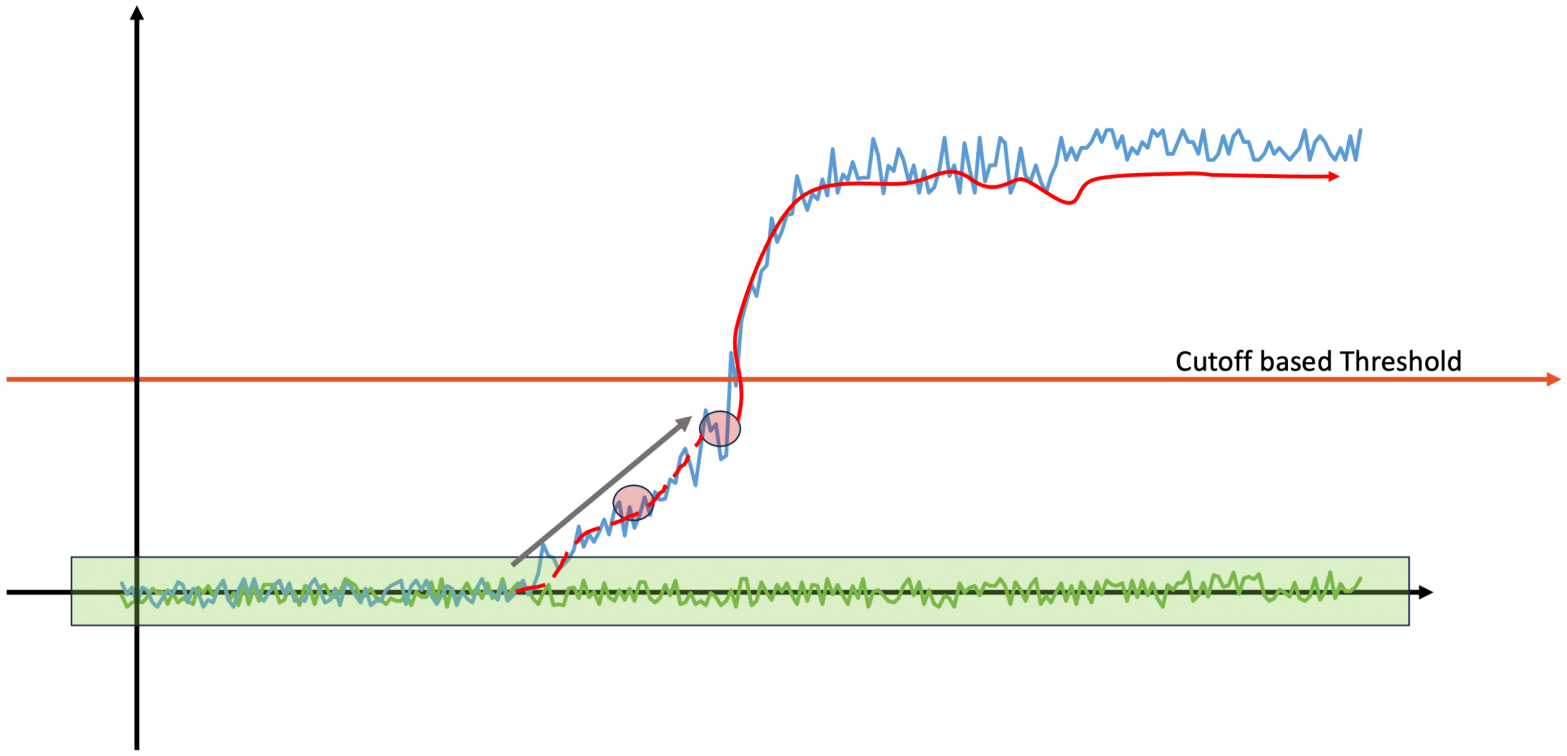
Linear approximation and considerations for the pre-threshold vs full model.

However, this framework does not aim to model that process but zooms in a small area of that process with an awareness of the start and end points. The starting point of this evaluation is the deviation away from the normal physiology, or as the model conceptualizes, the physiological plane. The endpoint is the crossing over of the diagnostic boundary. When the classic decision boundary is already breached, the clinician has proof of worsening, so the geometric framework does not serve any further diagnostic advantage. The patient can be flagged by either threshold-based or drift-based methods at that point in time. Linear approximation of what is possibly still a small part of the trajectory may be both mathematically acceptable and clinically translatable. Fig 9 demonstrates this concept.

The full curve is nonlinear and stochastic (blue). The dashed red line represents the actual disease trajectory within the noise-to-threshold zone, which can be locally approximated using linear assumptions. This model is not designed to fit the entire disease course but to identify early directional drift (gray arrow) away from the physiological corridor (green band). Later progression may require alternate modeling approaches, such as polynomial fitting (solid red line).

For example, imagine a cornea that starts to develop keratoconus beginning at 45D (when it was structurally normal and stable) and ends at 75D (when there is an advanced, end-stage disease). The change from 45D to hitting the statistical threshold at 47.4 D is a smaller part of that curve and the endpoints can be approximated. A clinician is also interested in knowing the trend (for example, a change from 45 to 46.5 D) on a more practical basis rather than knowing if a complex polynomial equation can map this change. That style of fitting, as we have previously demonstrated, becomes more useful in mapping the entire disease range, from normal through early to advanced [73]. From a more methodological perspective, this idea of linear regression is derived from the practice of segmented regression analysis used to evaluate changes in interrupted time series [97].

The visualization of the start point perhaps needs more explanation: it is the deviation away from normal in a significant, biologically plausible manner. This uses two concepts: normal noise envelope and angular alignment (cosine) with the disease process.

As discussed earlier, the noise in a subject-tester-instrument-interpretation environment can be accounted for using the intra-measurement standard deviation. This extends from the classic work of Bland and Altman [98]. This is a usual practice in many clinical domains, especially ophthalmology. Multiple previous repeatability and reliability studies have used the intra-measurement standard deviation (Sw) and its derived parameter the coefficient of repeatability (CR), especially for normal subjects [44,46,98–104] Again, reference change values (RCV) used for laboratory measurements are conceptually similar [31]. There is an interest in using a log-normal spread for reference change value (RCV) and this model can be customized based on the set of tests it is being applied to in the future [105]. For this study, we retained the CR at 2.77 Sw, per the Gaussian assumption. Using the Mahalanobis distance-based metric as an alternative is very promising, especially when we would deal with ≥3-dimensional testing, or we are forced to use correlated variables. Our future studies will explore this more in Python based platforms.

When measuring multiple variables, measures of confidence can be treated as orthogonal scalars creating a vector, as has been demonstrated previously [106,107]. This is generally derived from pooled data. However, to incorporate the real-world noisiness we calculated the individual vectors for Sw between the two parameters, accounting for a creeped-in covariance, and later scaled them by 2.77 to construct what we term the “noise fog envelope”. Like the rest of our model, this is also scalable into higher dimensions.

We deliberately avoided pooling noise metrics till later steps. This method retains the option of customization for a specific subject where the noise fog envelope can be calibrated based on their noted inter- measurement standard deviations(Sw) [42]. Once this envelope is modeled, it is supposed to be the upper limit of normal difference in measurement seen and therefore it was used to scale the change noted in the subject being tested. Classically, noise ellipses have been used as comparative tools (e.g., for inter and intra-device agreement). However, we have previously demonstrated their potential as a thresholding tool for clinical decision-making for cross-linking in progressive keratoconus [66]. The current metric is an extension of that concept.

Parameter values can change due to non-pathological reasons or due to a different pathology. For example, for a patient being evaluated for keratoconus, a tight contact lens can create a corneal warpage or scarring can cause corneal thinning. However, warpage will not induce significant corneal stromal thinning (can cause focal epithelial thickening) and corneal scarring will generally cause flattening [4,108]. So, the directionality of the pathological process becomes relevant (In our keratoconus example- corneal thinning and corneal steepening is the expected combination over time).

So, we need a method to note if the change is more than physiological noise AND is in the direction of the progression of pathology (alignment with the disease vector). This is the purpose of the combined metric, the Composite Drift Score (CDS). It combines both the magnitude and the directionality and thereby creates a potential metric to evaluate the change.

**Fig 10:**
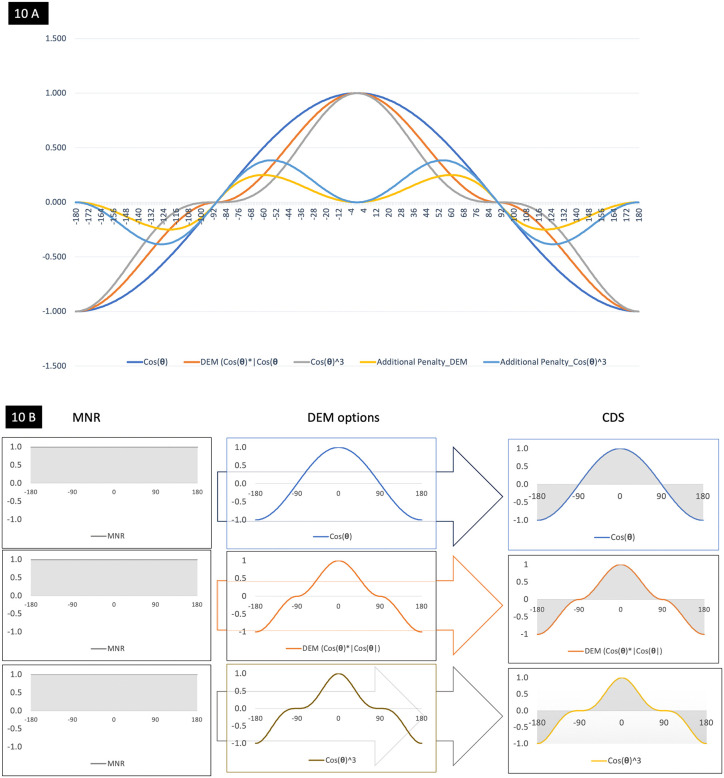
Comparison of waveforms of Angular Weighting Functions: Raw cosine (Cos(θ)), current Directional Emphasis Multiplier (DEM = Cos(θ)·|Cos(θ)|)) and a steeper cubed cosine (Cos^3^(θ)). The additional penalty values show the additional reduction each of two functions (DEM and Cubed cosine) imposes relative to Raw cosine. **Effect of Angular Weighting on CDS Magnitude.** Left: Constant magnitude-to-noise ratio (MNR). Center: Angular weighting functions: Cos(θ), DEM, and Cos^3^(θ). Right: Product of MNR and Angular function defines CDS magnitude (gray shaded area under curve).

A CDS threshold of 1 requires both conditions to be met simultaneously, the subject's drift must exceed the physiological noise boundary (MNR > 1) and must be directionally aligned with the disease vector (DEM > 0). Neither condition alone is sufficient, which is what gives the combined score its specificity. The raw cosine was replaced by the signed cosine-square (DEM = cos ϕ· |cos ϕ|) because it quadratically rewards strong alignment while progressively penalizing misalignment, without requiring an arbitrary cutoff at a particular angle(or degree of misalignment). This function is customizable. Steeper alternatives such as cos³(ϕ) increase directional specificity at the cost of sensitivity, as illustrated in Figs 10a and 10b. The operating properties of different weighting functions and their effect on CDS across the full angular range are shown there and are not re-derived here.

Further optimization of directional weighting, phenotype-specific modeling, and performance tuning across noisy datasets will be addressed in subsequent work.

A practical implementation of the CDS framework would follow a staged pathway. In the calibration stage, a disease-specific reference dataset is assembled from routine clinical records: stable normal subjects provide the noise scalar η and physiological plane, while confirmed progressive early-disease subjects provide the canonical disease vector D. These steps require two routinely measured variables and standard repeatability data already available in most clinical domains, for example, Pentacam-derived Kmax and TCT in corneal practice, or eGFR and urine albumin-to-creatinine ratio in nephrology [63,109]. The calibration dataset can be updated periodically as institutional data accumulates, analogous to how laboratory reference intervals are re-derived over time [35].

At the individual patient level during routine follow-up, the framework produces a single unitless output: CDS below 1.0 indicates that observed change is either within physiological noise or not aligned with the disease direction; CDS at or above 1.0 indicates supra-noise, disease-aligned drift warranting closer surveillance. Critically, CDS functions as a triage layer upstream of existing diagnostic workflows rather than as a standalone diagnostic, analogous to how rising biomarker velocity prompts further evaluation even when the absolute value remains below the diagnostic threshold [110–112]. Conversely, a persistently low CDS in a patient with borderline absolute values could provide quantitative reassurance and support longer surveillance intervals.

Several implementation considerations merit acknowledgement. The framework requires a minimum of two longitudinal visits and cannot operate on a single cross-sectional measurement. Calibration cohorts must be representative of the target population in terms of age, ethnicity, and measurement device. The CDS threshold of 1.0 is a mathematically motivated starting point; optimal thresholds for specific clinical applications will require ROC analysis in real-world datasets. The assumptions and boundary conditions under which this framework may underperform are discussed in below.

This study has several limitations inherent to its exploratory, proof-of-concept design. The framework was evaluated on synthetic data generated under controlled assumptions; performance in real-world datasets with irregular follow-up intervals, missing visits, and population heterogeneity remains to be established in future work. The current implementation uses two variables; while the Mahalanobis extension was derived and showed consistent trends, systematic comparison with the Euclidean approach in higher-dimensional or strongly correlated settings is reserved for subsequent studies. The canonical disease vector assumes a dominant directional trajectory; diseases with distinct phenotypic subtypes following divergent progression paths may benefit from subtype-specific vectors, which the framework can accommodate through separate calibration cohorts. The linear approximation within the noise-to-threshold window is intentional and appropriate for this narrow zone, though its adequacy for diseases with abrupt or stepwise transitions would need to be evaluated individually. The noise scalar η is derived from pooled normal data and assumes broadly comparable measurement variability across subjects; personalized noise calibration, which the framework supports, may improve performance in heterogeneous clinical settings. Finally, while the mathematical structure is domain-agnostic, transferability beyond the keratoconus model used here requires independent calibration and validation in each clinical setting.

## Conclusion

We hypothesized a 2-plane disease vs normal model, and conceptualized a mathematical framework based on it. Then we constructed an index system, CDS (= DEM x MNR) to with the eventual target to flag early change within the subthreshold range in synthetic data. This metric and its subcomponents were able to demonstrate similar trends in progressive early disease and progressive pre-threshold groups. This suggests that with more studies, rigorous evaluation, and iterations it may be able to mathematically map its intended overarching goal: a unitless ratio based intuitive method to denote disease directional change in the pre-threshold group and therefore have impact in early detection and management of a subset of chronic diseases.

Threshold based tests are agnostic to pre-threshold movement. Therefore, the next step is to compare these metrics in unlabeled data and evaluate their performance and lead time compared to threshold-based cutoffs. This early-stage simulation study sets up ground to evaluate this metric in our next work on stress testing this on highly stochastic data, then conceptualizing case-based scenarios, leading to possible multicentric/ collaborative work in real life situations.

## Supporting information

S1 Appendix Full mathematical derivations of the geometric drift framework, including complete derivations of normalization equations (Eqs. 2a–2c), physiological and pathological plane construction (Eqs. 3–5), noise scalar (Eqs. 6a–6f), age-related drift (Eqs. 6g–6i), disease vector (Eqs. 6j–6q), subject drift vector (Eqs. 6r–6t), and Mahalanobis distance framework (Eqs. 8a–8i). (DOCX)

S2 Appendix Synthetic dataset generation methodology: group-specific parameter distributions, randomisation logic, Gaussian noise specifications, seed values, and Mahalanobis distance computation pipeline. (DOCX)

S3 Appendix Excel dataset containing the full synthetic data for all four groups (NS, ED_S, ED_P, PT_P; n = 1000 each), including raw variables, normalised variables, computed drift vectors, and CR-based and Mahalanobis-based metrics across all follow-up timepoints. Publicly available at https://doi.org/10.17605/OSF.IO/2JNQF
(XLSX)

S4 Appendix Visual representation of the framework construction steps (panels A–K): raw data scatter, directional alignment, plane construction, normalisation, disease vector derivation, noise envelope, and subject vector evaluation. (PDF)
