# Supplementary material for: Directional drift in biologically meaningful vector planes: A proposed geometric framework for early detection of subthreshold disease
Source: PLoS One. 2026 Jul 30;21(7):e0353723. doi: 10.1371/journal.pone.0353723 (PMC13423176; doi:10.1371/journal.pone.0353723)
Supplement: S2 Appendix — (DOCX) [file pone.0353723.s002.docx]

**Supplementary Appendix B**

**Synthetic Dataset Generation: Parameters, Logic, and Reproducibility**

This appendix provides the complete data generation methodology for the synthetic dataset used in this study, including group-specific parameter distributions, randomisation logic, Gaussian noise specifications, and progression rules. All parameters were derived from published clinical data [Kreps EO et al., Am J Ophthalmol 2020; Kosekahya P et al., Eye Contact Lens 2019]. The dataset was generated in Microsoft Excel using structured NORM.INV-based sampling. Random number generation has been replaced with pasted values in the deposited file to ensure reproducibility across versions; the original logic formulae are preserved in the Excel Logic sheet and can be used to regenerate the data with a fresh random seed. The full dataset and logic file are deposited as Appendix 1 (Supplementary Information).

**1. Variable Definitions and Directional Alignment**

**Variable x:** Steepest keratometry (Kmax), units: Diopters. Direction of pathological worsening: increasing (+1).

**Variable y:** Thinnest corneal thickness (TCT), units: microns. Direction of pathological worsening: decreasing (−1).

Both variables were directionally aligned prior to normalisation so that increasing values in both x(n) and y(n) consistently indicate greater pathological deviation, regardless of raw variable direction (Methods Section 2; Supplementary Appendix A, Eqs. 2b–2c).

**2. Physiological Reference Range and Dataset-Level Parameters**

The following values, derived from the Normal Stable (NS) group, were used to define the physiological plane, noise scalar, and canonical disease vector:

| **Parameter** | **Value** |
| --- | --- |
| Largest normal Kmax (x_max) | 46.32 D |
| Smallest normal Kmax (x_min) | 41.08 D |
| Physiological range x (R_phy_x) | 5.24 D |
| Largest normal TCT (y_max) | 596.11 µm |
| Smallest normal TCT (y_min) | 495.06 µm |
| Physiological range y (R_phy_y) | 101.04 µm |
| Pooled noise scalar η (CR-based) | 0.1112 |
| Disease vector angle θ_D | 41.95° |
| Disease vector mean Δx(d) | 0.2145 (SD 0.129) |
| Disease vector mean Δy(d) | 0.1928 (SD 0.119) |

*Disease vector parameters are normalised mean change values per 6-month interval, pooled from the ED_P group across t1–t5.*

**3. Group-Specific Randomisation Rules**

The table below documents the complete randomisation logic for all four groups across baseline and follow-up timepoints. Baseline values were drawn from truncated normal distributions (clipped at 1st–99th percentiles). Follow-up values incorporated group-specific noise and, where applicable, directional drift added to the preceding timepoint value (cumulative model).

| **Normal Stable (NS)** | | | | |
| --- | --- | --- | --- | --- |
|  | Step 1 | X_t0 – Baseline Kmax | Simulate from N(43.7, 1.0), clipped between 1st and 99th percentiles | MIN(MAX(NORM.INV(RAND(), 43.7, 1), NORM.INV(0.01, 43.7, 1)), NORM.INV(0.99, 43.7, 1)) |
|  | Step 2 | Y_t0 – Baseline TCT | Simulate from N(544.4, 18.6), clipped between 1st and 99th percentiles | MIN(MAX(NORM.INV(RAND(), 544.4, 18.6), NORM.INV(0.01, 544.4, 18.6)), NORM.INV(0.99, 544.4, 18.6)) |
|  | Step 3 | X_t1 to X_t5 | Add noise only: each step = X_t0 + N(0, 0.17) | X_t0 + NORM.INV(RAND(), 0, 0.17) ← repeat 4 more times |
|  | Step 4 | Y_t1 to Y_t5 | Add noise only: each step = Y_t0 + N(0, 3.04) | Y_t0 + NORM.INV(RAND(), 0, 3.04) ← repeat 4 more times |
| **Early Disease Stable (ED_S)** | | | | |
|  | Step 1 | X_t0 – Baseline Kmax | Simulate from N(48.8, 2.4), clipped between 1st and 99th percentiles | MIN(MAX(NORM.INV(RAND(), 48.8, 2.4), NORM.INV(0.01, 48.8, 2.4)), NORM.INV(0.99, 48.8, 2.4)) |
|  | Step 2 | Y_t0 – Baseline TCT | Simulate from N(498.3, 26.5), clipped as above | MIN(MAX(NORM.INV(RAND(), 498.3, 26.5), NORM.INV(0.01, 498.3, 26.5)), NORM.INV(0.99, 498.3, 26.5)) |
|  | Step 3 | X_t1 to X_t5 | Add noise only: mean 0, SD = 0.22 | X_t0 + NORM.INV(RAND(), 0, 0.22) ← repeat 4 more times |
|  | Step 4 | Y_t1 to Y_t5 | Add noise only: mean 0, SD = 4.08 | Y_t0 + NORM.INV(RAND(), 0, 4.08) ← repeat 4 more times |
| **Early Disease Progressive (ED_P)** | | | | |
|  | Step 1 | X_t0 – Baseline Kmax | Simulate from N(48.8, 2.4), clipped between 1st and 99th percentiles | MIN(MAX(NORM.INV(RAND(), 48.8, 2.4), NORM.INV(0.01, 48.8, 2.4)), NORM.INV(0.99, 48.8, 2.4)) |
|  | Step 2 | Y_t0 – Baseline TCT | Simulate from N(498.3, 26.5), clipped as above | MIN(MAX(NORM.INV(RAND(), 498.3, 26.5), NORM.INV(0.01, 498.3, 26.5)), NORM.INV(0.99, 498.3, 26.5)) |
|  | Step 3 | X_t1 to X_t5 | Add: 0 ± 0.22 (noise) + 0.075 ± 0.025 (drift) to previous X | X_t(n-1) + NORM.INV(RAND(), 0, 0.22) + NORM.INV(RAND(), 0.075, 0.025) ← repeat 4 more times |
|  | Step 4 | Y_t1 to Y_t5 | Add: 0 ± 4.08 (noise) + (-1.3 ± 0.25) (drift) to previous Y | Y_t(n-1) + NORM.INV(RAND(), 0, 4.08) + NORM.INV(RAND(), -1.3, 0.25) ← repeat 4 more times |
| **Pre-threshold Progressive (PT_P)** | | | | |
|  | Step 1 | X_t0 – Baseline Kmax | Simulate from N(43.7, 1.0), clipped between 1st and 99th percentiles (same as NS) | MIN(MAX(NORM.INV(RAND(), 43.7, 1), NORM.INV(0.01, 43.7, 1)), NORM.INV(0.99, 43.7, 1)) |
|  | Step 2 | Y_t0 – Baseline TCT | Simulate from N(544.4, 18.6), clipped as above (same as NS) | MIN(MAX(NORM.INV(RAND(), 544.4, 18.6), NORM.INV(0.01, 544.4, 18.6)), NORM.INV(0.99, 544.4, 18.6)) |
|  | Step 3 | X_t1 to X_t5 | Add: 0 ± 0.17 (noise) + 0.064 ± 0.02 (drift) to previous X | X_t(n-1) + NORM.INV(RAND(), 0, 0.17) + NORM.INV(RAND(), 0.064, 0.02) ← repeat 4 more times |
|  | Step 4 | Y_t1 to Y_t5 | Add: 0 ± 3.04 (noise) + (-0.75 ± 0.2) (drift) to previous Y | Y_t(n-1) + NORM.INV(RAND(), 0, 3.04) + NORM.INV(RAND(), -0.75, 0.2) ← repeat 4 more times |

Notes: *[a] Kreps EO et al. Repeatability of the Pentacam HR in Various Grades of Keratoconus. Am J Ophthalmol.* 2020;219:154–162. *[b] Kosekahya P et al. Longitudinal Evaluation of the Progression of Keratoconus Using a Novel Progression Display. Eye Contact Lens.* 2019;45(5):324–330. PT_P drift rates set at approximately 80% of ED_P values, reflecting the pre-diagnostic window where direct empirical progression data are unavailable.

**4. Reproducibility and Seed Values**

The synthetic dataset was generated using Microsoft Excel's NORM.INV(RAND(), μ, σ) function with clipping applied at the 1st and 99th percentiles for all baseline values. To ensure reproducibility, random number generation has been replaced with pasted values in the deposited Appendix C file. The Excel Logic sheet retains the original generating formulae, allowing the dataset to be regenerated with a fresh random seed or transposed to Python/R using identical parameter distributions.

The specific values in the deposited dataset represent a single realization of the stochastic process. All key findings :group separation, CDS trends, and directional alignment :were confirmed to be stable across multiple independent realizations during development, consistent with the large sample size (n = 1000 per group) and the signal-to-noise ratio of the progressive groups.

**5. Mahalanobis Distance Calculation: Step-by-Step Logic**

The following table documents the complete computational steps for the Mahalanobis distance-based metrics (MNR(MD) and CDS(MD)), with cell references to the deposited Appendix C Excel file:

| **Step** | **Action** | **Formula / Explanation** | **Remarks** |
| --- | --- | --- | --- |
| **5a** | Stack normalised ΔX and ΔY vectors for all subjects (follow-up − baseline) | Use INDEX logic to create stacked matrix of (ΔX, ΔY) for all follow-ups | See 'Stacked' sheet in Appendix 1 |
| **5b** | Compute covariance matrix from stacked ΔX and ΔY | VAR.S, COVARIANCE.S → elements a (Var_x = 0.001060), b (Cov = −8.55×10⁻⁶), d (Var_y = 0.000873) | Reference sheet, cells B39–B41 |
| **5c** | Compute determinant of covariance matrix | det = ad − b² = 9.256×10⁻⁷ | Reference sheet, cell B42 |
| **5d** | Compute inverse covariance matrix Σ⁻¹ | Σ⁻¹(1,1) = d/det = 943.74; Σ⁻¹(1,2) = Σ⁻¹(2,1) = −b/det = 9.23; Σ⁻¹(2,2) = a/det = 1144.93 | Reference sheet, cells B44–B47 |
| **5e** | For each subject/timepoint, compute ΔX and ΔY relative to baseline | ΔX = X_t(n) − X_t0; ΔY = Y_t(n) − Y_t0 | Delta columns in each group sheet |
| **5f** | Compute Mahalanobis distance | MD = √( (ΔX·a + ΔY·b)·ΔX + (ΔX·b + ΔY·d)·ΔY ) | See cell O45 in Reference sheet for full expansion |
| **5g** | Results stored | MD values in columns MahaD_T1 to MahaD_T5 in each group sheet |  |
| **5h** | Compute MNR(MD) | MNR(MD) = MD / 2.45 [critical threshold at α=0.05, df=2: √χ²₀.₀₅ = √5.99 ≈ 2.45] | Columns MNR_Maha_T1 to T5 |
| **5i** | Compute CDS(MD) | CDS(MD) = DEM × MNR(MD) | Columns CDS_Maha_T1 to T5 |

*The near-zero covariance between Δx(n) and Δy(n) (b = −8.55×10⁻⁶) confirms that Kmax and TCT contribute nearly orthogonally to the noise space in this 2D dataset. This validates the use of the Euclidean CR-based approach as the primary metric for this study. The Mahalanobis framework is recommended for future implementations with ≥3 variables or substantially correlated variable pairs.*

**6. Data Availability**

The complete synthetic dataset and data generation logic (Appendix C: Excel sheet) and the processing steps visual representation (Appendix D: PDF) are provided as supplementary files and are deposited in a public repository in accordance with PLOS ONE data sharing policy. All data are available without restriction.
