# Supplementary material for: Directional drift in biologically meaningful vector planes: A proposed geometric framework for early detection of subthreshold disease
Source: PLoS One. 2026 Jul 30;21(7):e0353723. doi: 10.1371/journal.pone.0353723 (PMC13423176; doi:10.1371/journal.pone.0353723)
Supplement: S4 Appendix — (PDF) [file pone.0353723.s004.pdf]

A

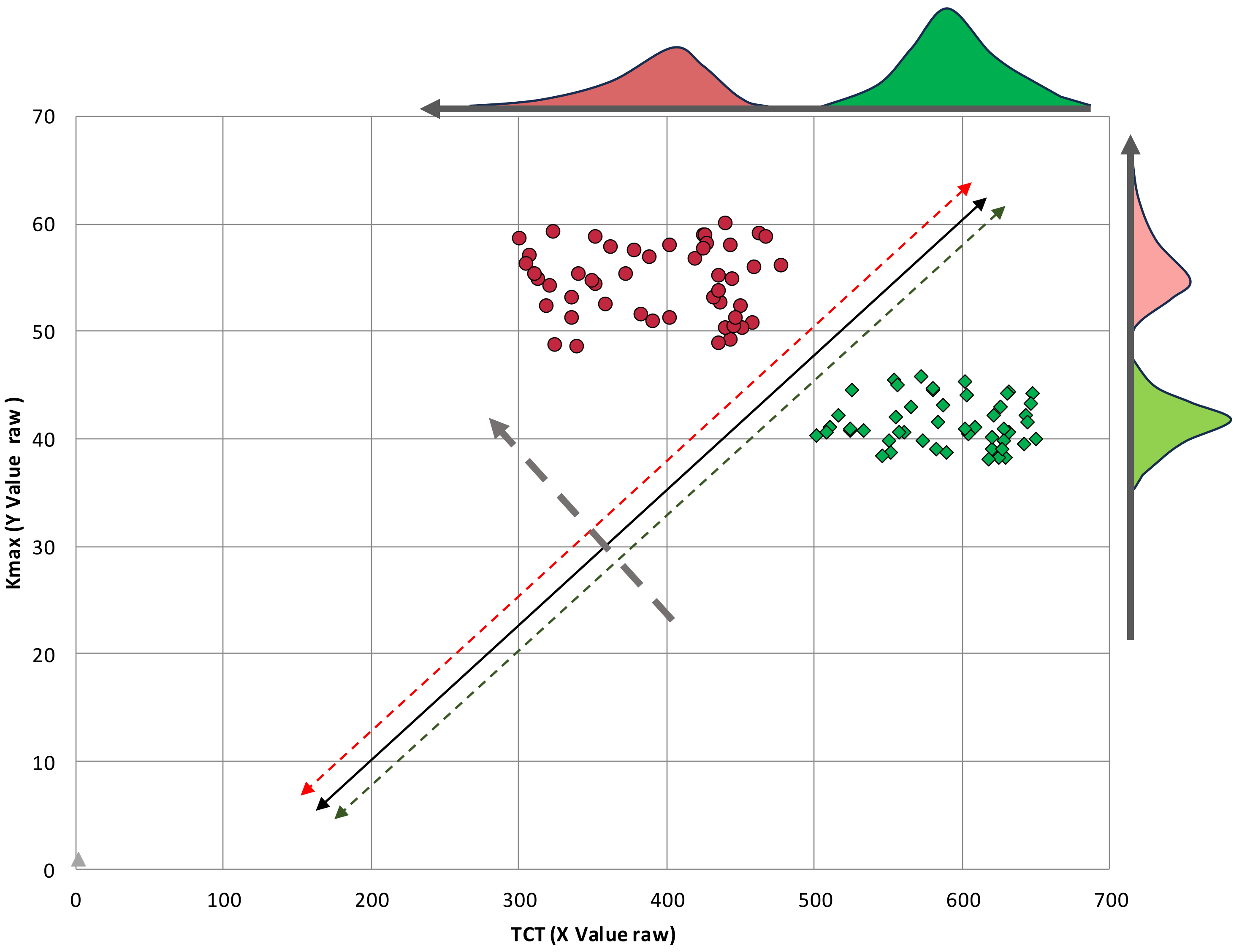

Raw scatterplot of Kmax and TCT values, with distribution plots as overlays. Due to differing units and directions, direct algebraic plotting can mislead interpretation. TCT has a larger numerical range (~350 microns) compared to Kmax (25D) and a different set of values seen (Kmax from 35 to 70 D and TCT from 300 to 650 microns), and the disease trajectories are inverse (TCT decreases, Kmax increases).

B

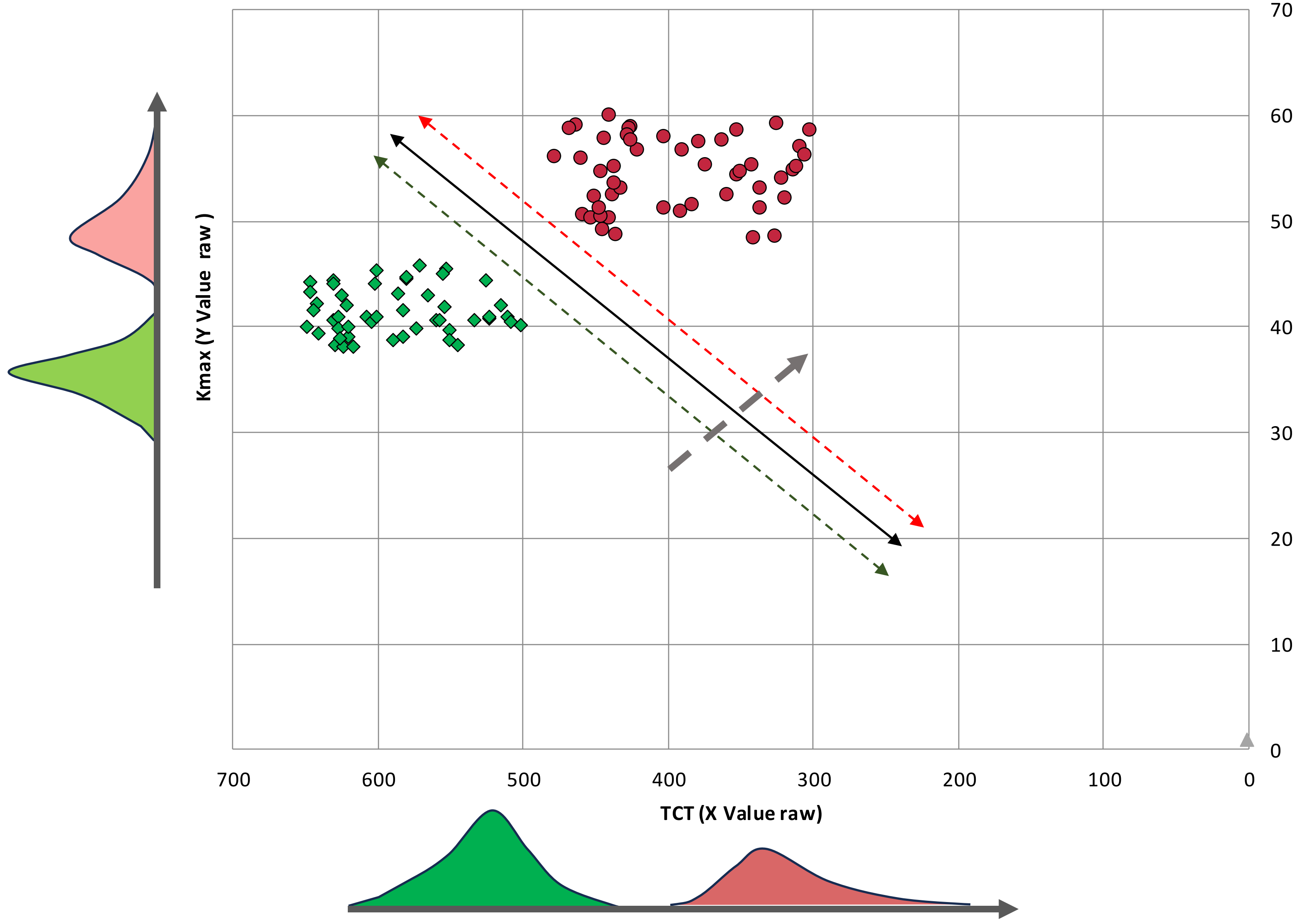

Axis inversion for TCT aligns it directionally with Kmax with the disease process. This step improves interpretability by mapping both variables consistently relative to disease progression. The scaling is still an issue.

Adjusting TCT and Kmax to the same direction improves interpretation

C

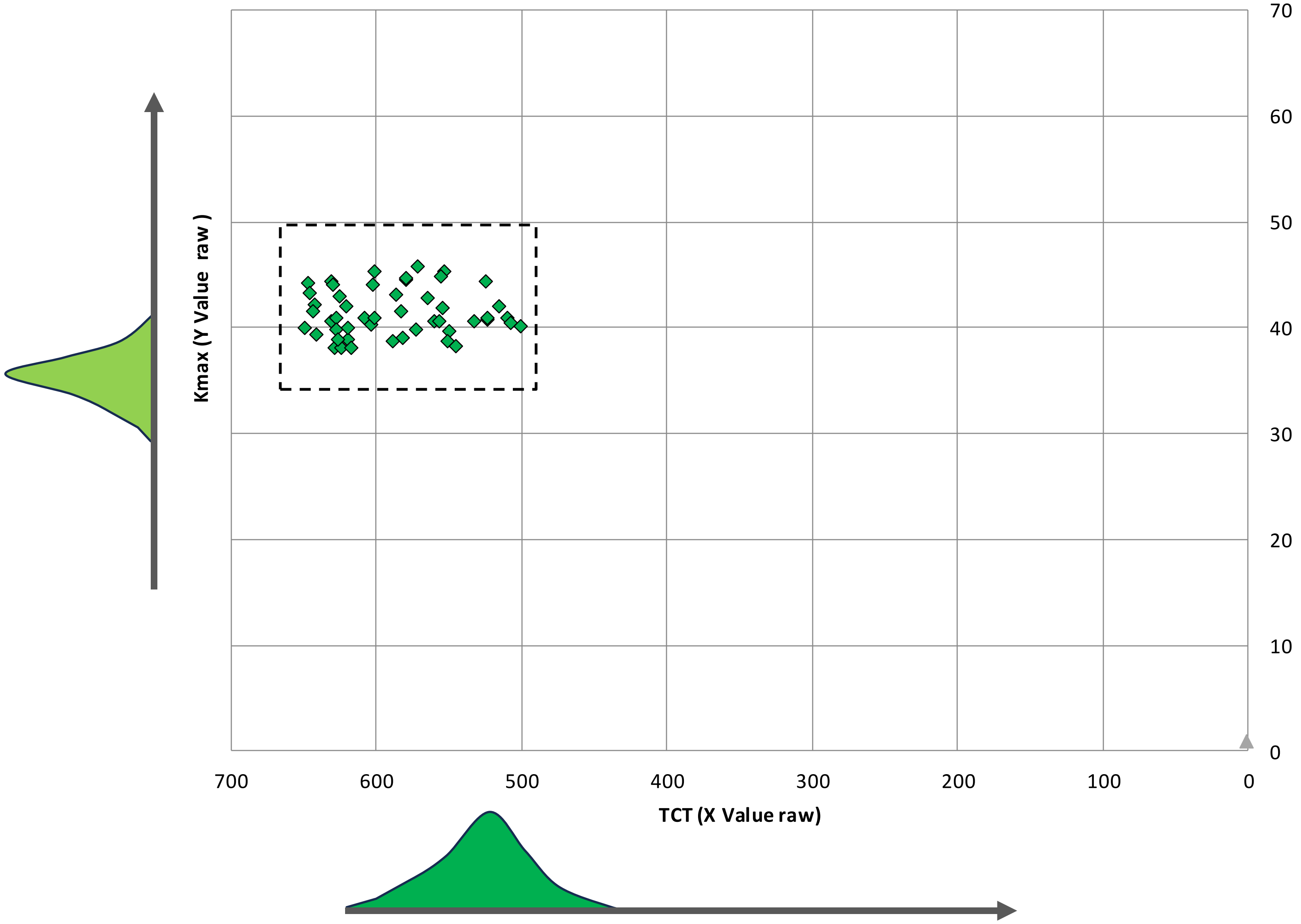

Creation of the planes: step 1 : consider the normal range of values, avoid borderline cases

D

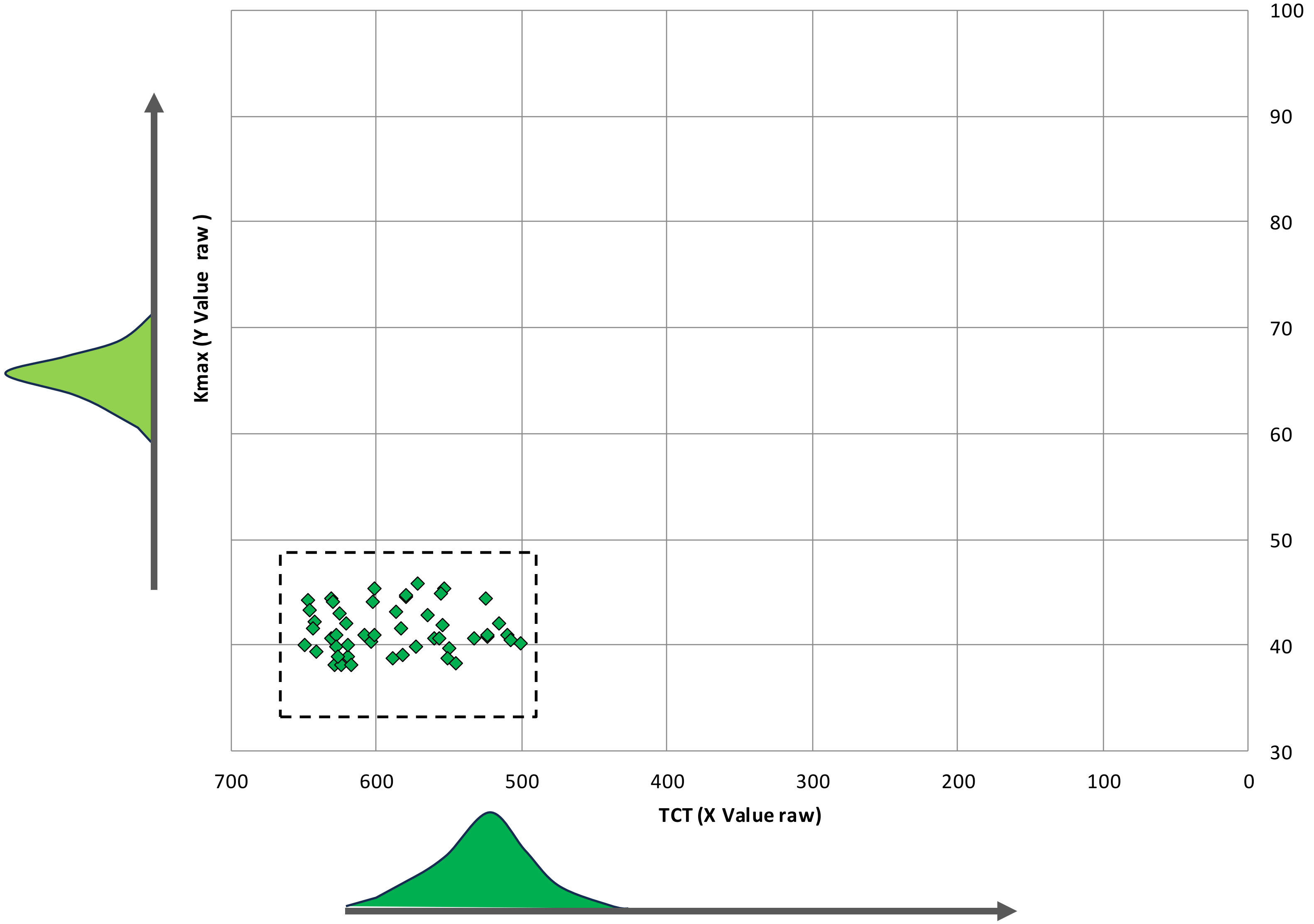

Creation of the planes: step 2 :
Appropriate adjustment of scales to create a left sided bounded quadrant

E

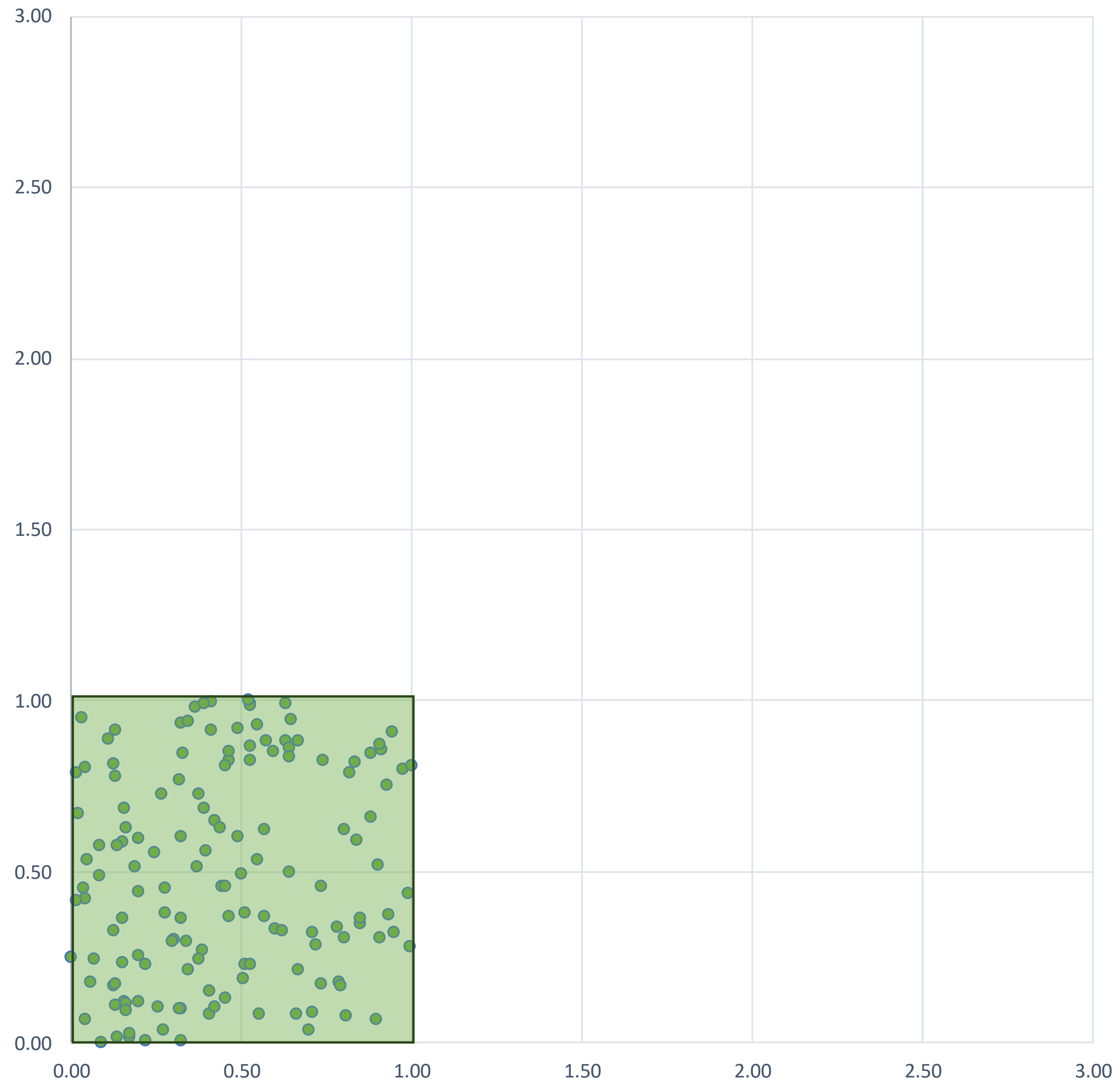

Normalized region
For the physiological values:
2 D representation of the physiological plane

F

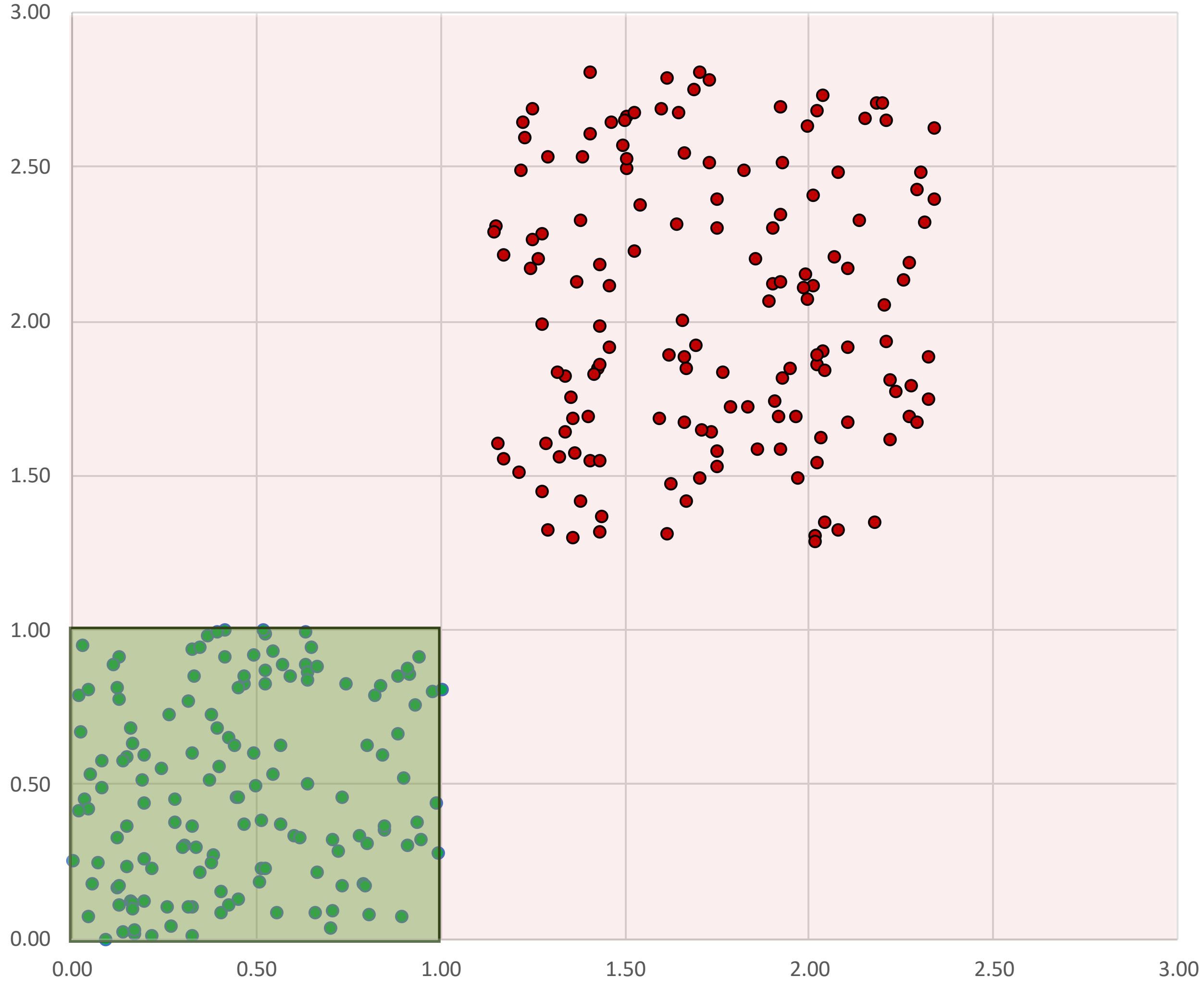

Normalized region
For the physiological values:
2 D representation of the physiological plane

Similar scaling used for the pathological Values: 2 D representation of the Pathological plane

G

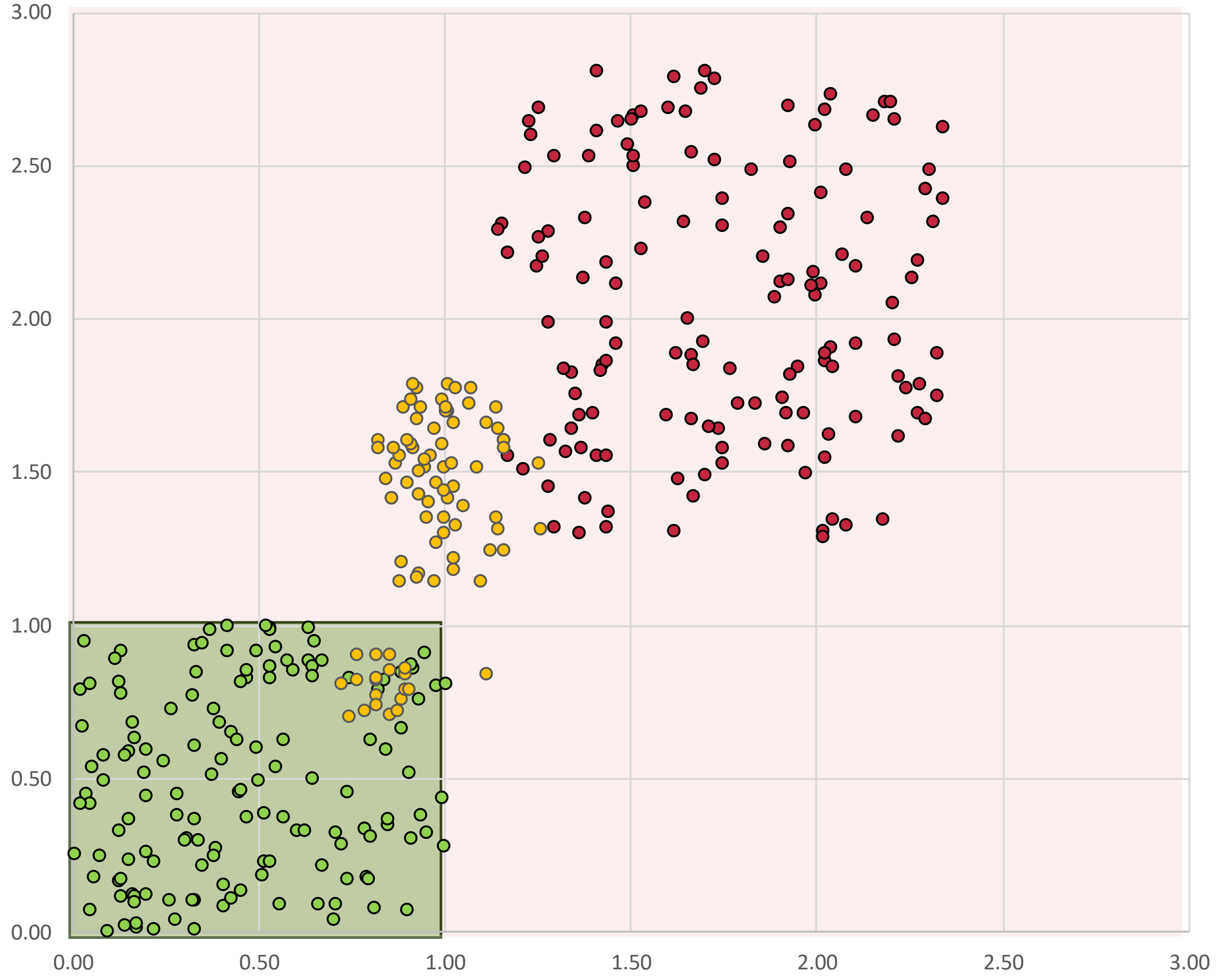

2D overlay still does not solve the problem
Of decision boundary and suspects (orange)
Seen in real world

H

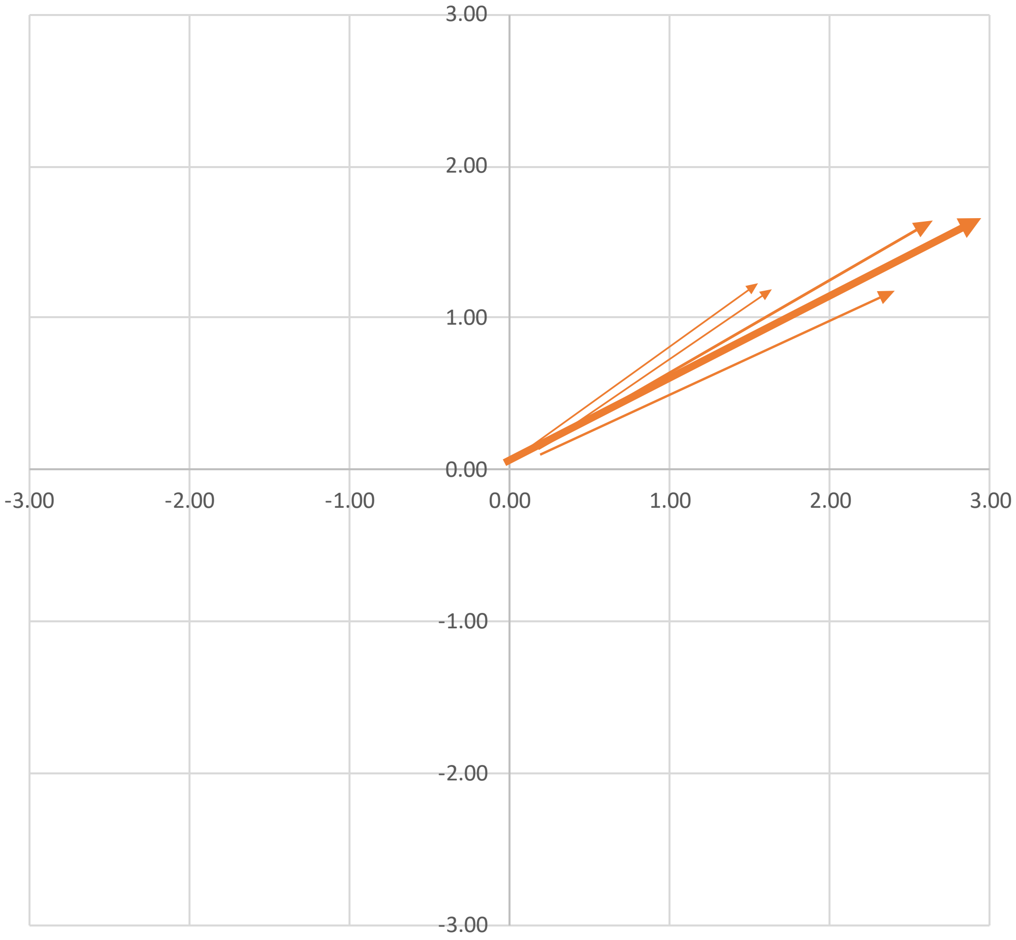

Disease direction ($\vec{D}$)

The canonical disease vector is calculated by pooling x- and y-coordinate changes from early progressive cases. This defines the reference direction for disease drift.

I

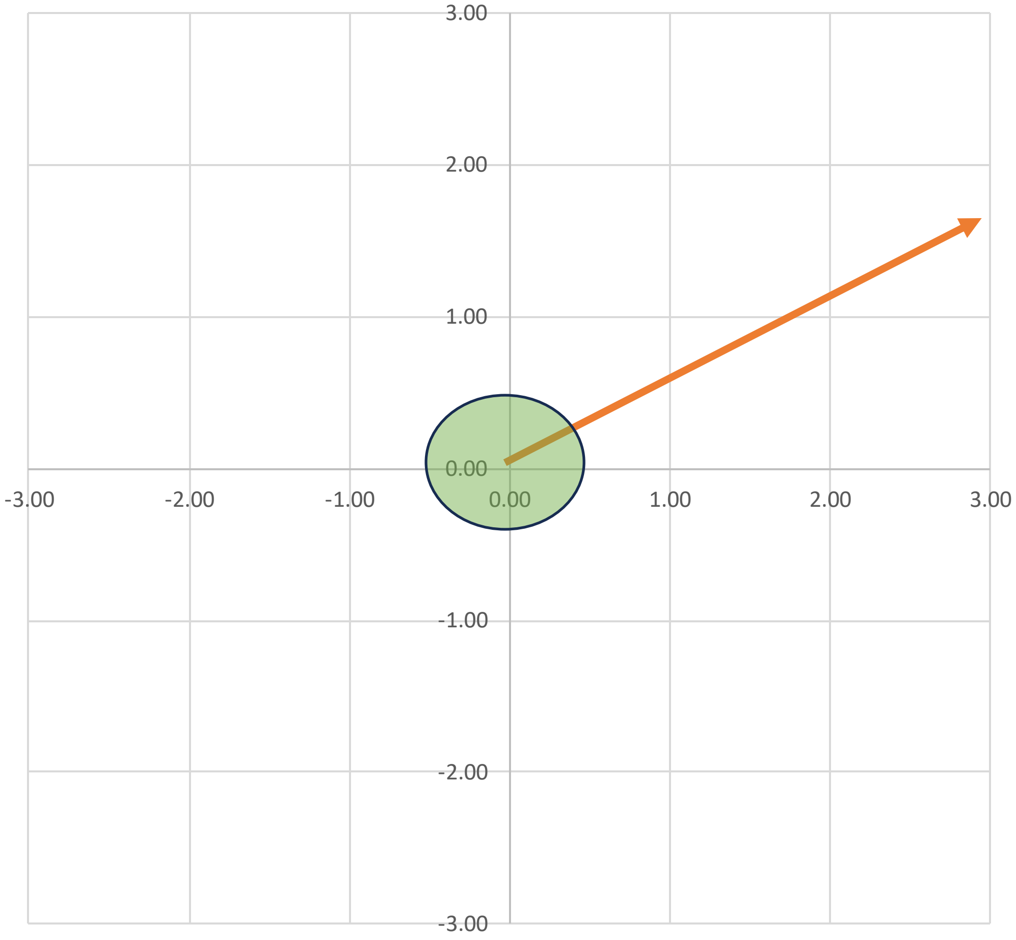

Disease direction ($\vec{D}$)
All patient vectors are compared to this canonical direction

J

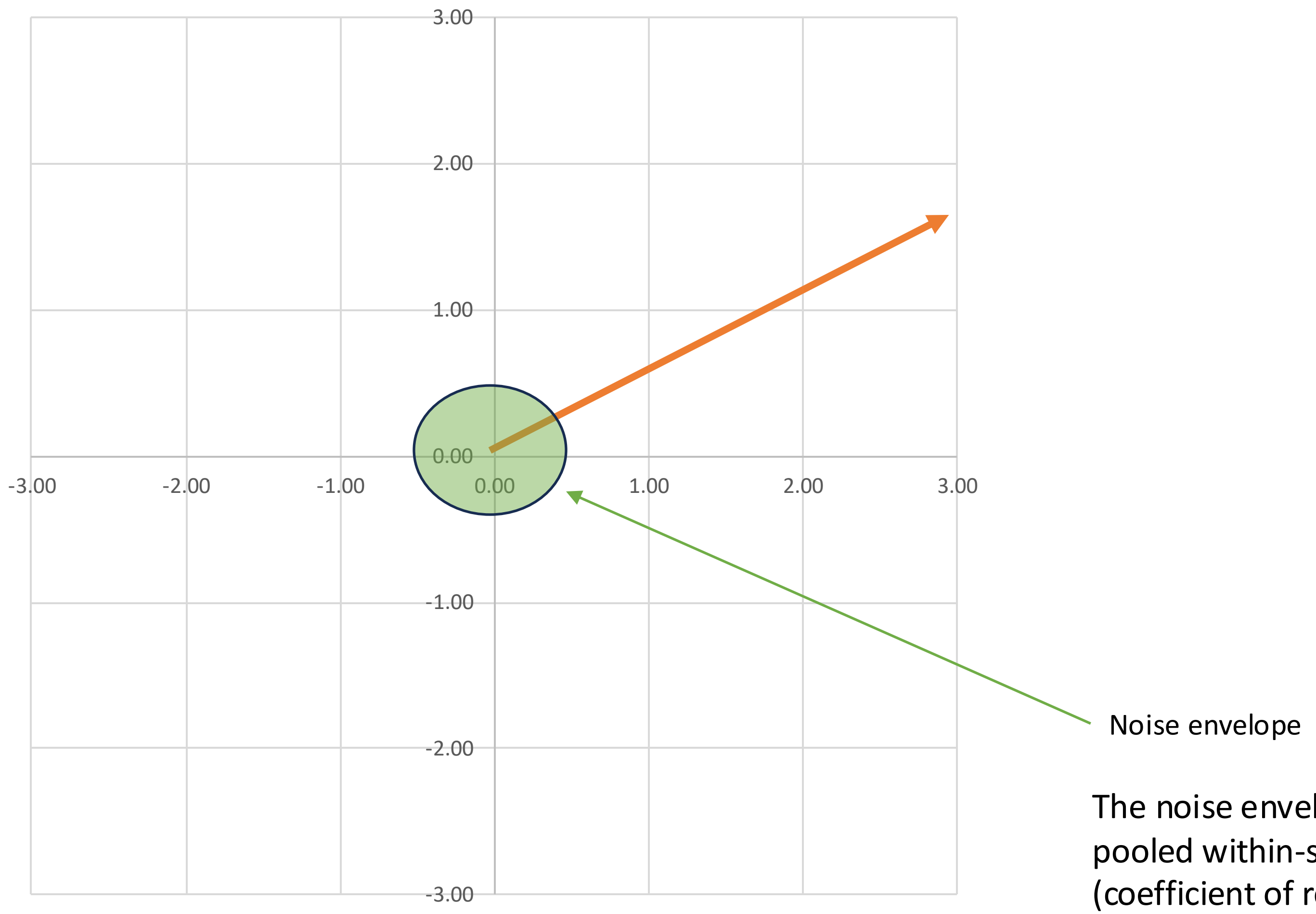

The noise envelope is derived from pooled within-subject variability (coefficient of repeatability) in the physiological group. It is visualized as boundary centered at origin

K

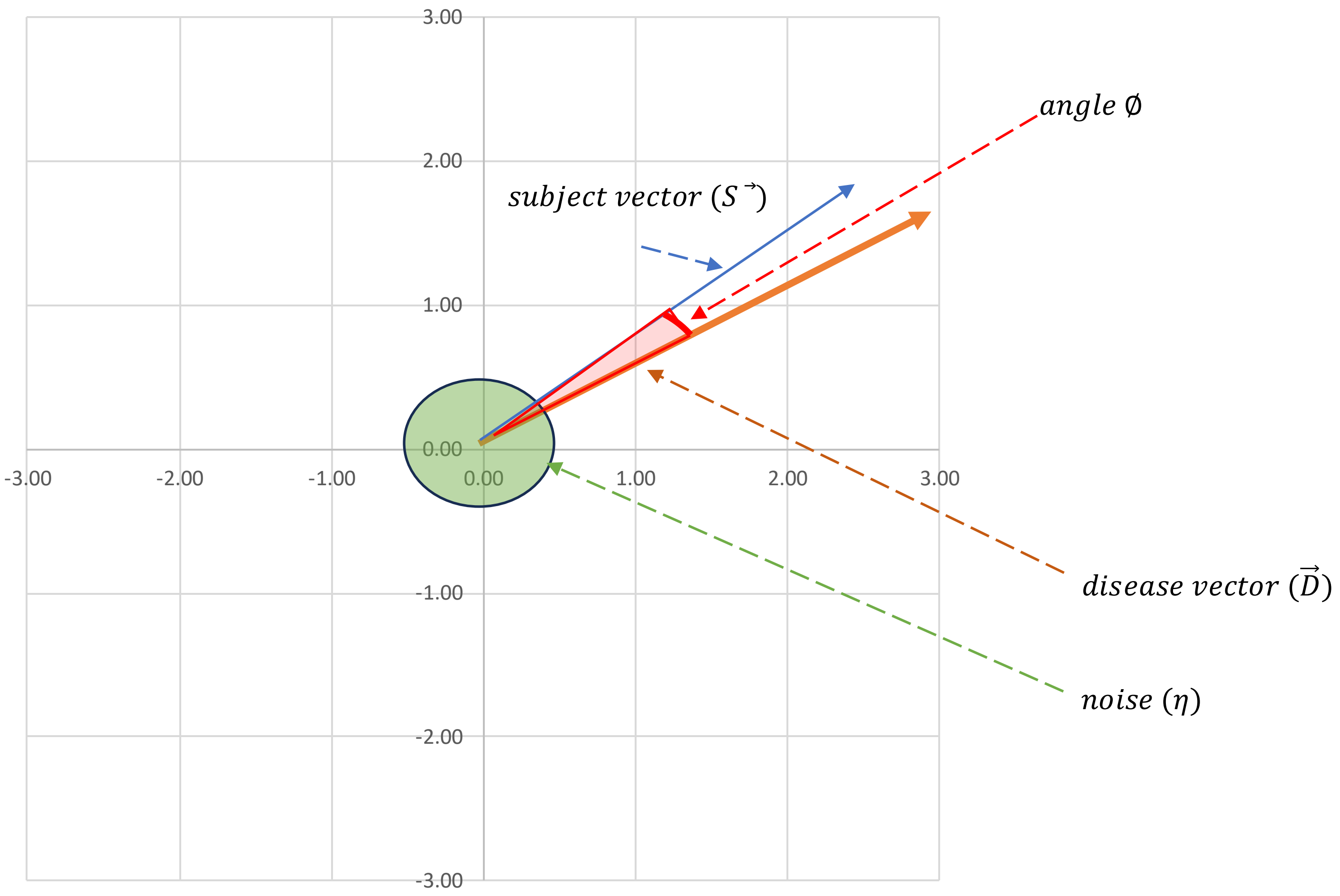

Each subject's drift vector is evaluated in terms of angular alignment with disease vector and ratio of its magnitude with noise
